# When taxonomy and biological control researchers unite: Species delimitation of *Eadya* parasitoids (Braconidae) and consequences for classical biological control of invasive paropsine pests of *Eucalyptus*

**DOI:** 10.1371/journal.pone.0201276

**Published:** 2018-08-16

**Authors:** Leanne Peixoto, Geoff R. Allen, Ryan D. Ridenbaugh, Stephen R. Quarrell, Toni M. Withers, Barbara J. Sharanowski

**Affiliations:** 1 University of Central Florida, Department of Biology, Orlando, FL, United States of Amrica; 2 Aarhus University, Department of Agroecology, Aarhus, Denmark; 3 Tasmanian Institute of Agriculture, University of Tasmania, Hobart, TAS, Australia; 4 Scion, Rotorua, New Zealand; Nanjing Agricultural University, CHINA

## Abstract

The invasive eucalyptus tortoise beetle, *Paropsis charybdis*, defoliates plantations of *Eucalyptus nitens* in New Zealand. Recent efforts to identify host specific biological control agents (parasitoids) from Tasmania, Australia, have focused on the larval parasitoid wasp, *Eadya paropsidis* (Braconidae), first described in 1978. In Tasmania, *Eadya* has been reared from *Paropsisterna agricola* (genus abbreviated *Pst*.), a smaller paropsine that feeds as a larva on juvenile rather than adult foliage of *Eucalyptus nitens*. To determine which of the many paropsine beetle hosts native to Tasmania are utilized by *E*. *paropsidis*, and to rule out the presence of cryptic species, a molecular phylogenetic approach was combined with host data from rearing experiments from multiple locations across six years. Sampling included 188 wasps and 94 beetles for molecular data alone. Two mitochondrial genes (*COI* and *Cytb*) and one nuclear gene (*28S*) were analyzed to assess the species limits in the parasitoid wasps. The mitochondrial genes were congruent in delimiting four separate phylogenetic species, all supported by morphological examinations of *Eadya* specimens collected throughout Tasmania. *Eadya paropsidis* was true to the type description, and was almost exclusively associated with *P*. *tasmanica*. A new cryptic species similar to *E*. *paropsidis*, *Eadya* sp. 3, was readily reared from *Pst*. *agricola* and *P*. *charybdis* from all sites and all years. *Eadya* sp. 3 represents the best candidate for biological control of *P*. *charybdis* and was determined as the species undergoing host range testing in New Zealand for its potential as a biological control agent. Another new species, *Eadya* sp. 1, was morphologically distinctive and attacked multiple hosts. The most common host was *Pst*. *variicollis*, but was also reared from *Pst*. *nobilitata* and *Pst*. *selmani*. *Eadya* sp. 1 may have potential for control against *Pst*. *variicollis*, a new incursion in New Zealand, and possibly *Pst*. *selmani* in Ireland. Our molecular data suggests that *Pst*. *variicollis* is in need of taxonomic revision and the geographic source of the beetle in New Zealand may not be Tasmania. *Eadya* sp. 2 was rarely collected and attacked *P*. *aegrota elliotti* and *P*. *charybdis*. Most species of *Eadya* present in Tasmania are not host specific to one beetle species alone, but demonstrate some host plasticity across the genera *Paropsisterna* and *Paropsis*. This study is an excellent example of collaborative phylogenetic and biological control research prior to the release of prospective biological control agents, and has important implications for the *Eucalyptus* industry worldwide.

## Introduction

Classical biological control of insects, involving the importation of a specialist parasitoid or predatory organism (agents) from the area of origin to control a pest (target), has proven to be an effective alternative to insecticide use for the control of numerous invasive species [1–3]. Classical biological control is now only considered when environmental safety concerns can be empirically evaluated. Success of classical biological control programs depends on ensuring: (1) an agent is sufficiently host specific to the pest to avoid significant non-target impacts; (2) a phenological match between the target and agent to facilitate efficient control and also prevent non-target impacts; and (3) the agent can survive and reproduce in the novel environment [4, 2, 5–7].

Cryptic species present another challenge for successful biological control [8–11]. Cryptic species complexes are typically comprised of a set of related species that are morphologically indistinguishable or difficult to diagnose based on morphology alone. As the majority of taxonomic works have been based on only morphological characters, cryptic species complexes typically include a set of undescribed species [12]. Molecular taxonomy has revealed numerous cryptic species complexes, particularly within insects [13–18]. If a prospective biological control agent is a part of a cryptic species complex, multiple species may end up being released causing non-target effects, or if the wrong species is released, control of the target pest may not occur [11, 7, 19]. Thus, it is critical to test for the presence of cryptic species by sampling specimens from a wide range of localities from the agent’s origin.

**Fig 1:**
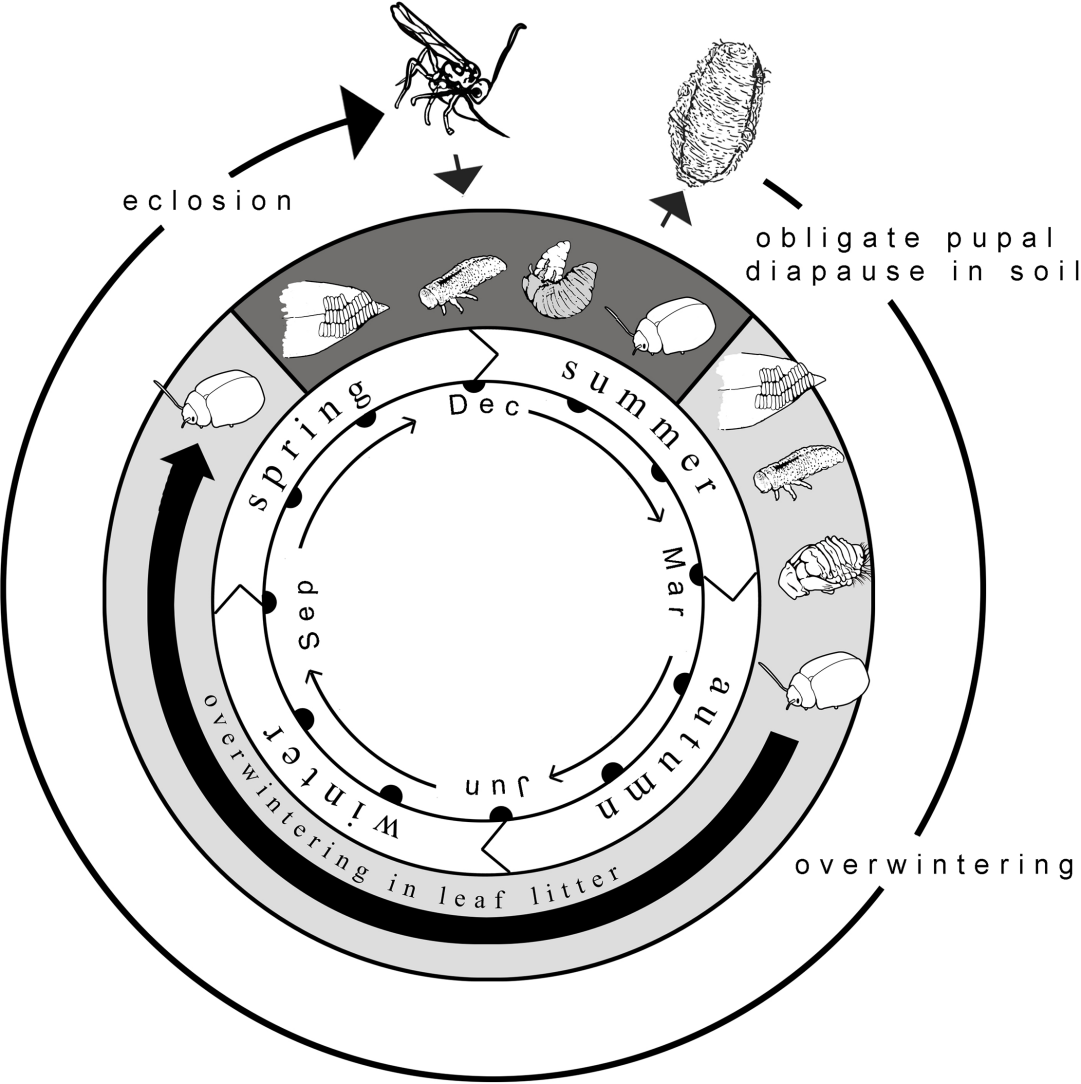
Generalized life cycle and phenological synchrony of *Eadya* in relation to the larval stages of its paropsine beetle hosts in Tasmania. Although there are two generations of paropsine beetles in Tasmania, *Eadya* are univoltine, attacking only the first (spring) generation (dark gray). *Eadya* are larval parasitoids, typically attacking 2^nd^ instar larvae and emerging from the prepupal stage. Despite a second generation of beetle hosts (light gray) available, *Eadya* undergo a ten month obligate pupal diapause period.

*Eucalyptus* plantations are an important wood and pulp fiber resource for the forestry industry in many countries in the world; however, the survival and expansion of these plantations in New Zealand and elsewhere are under threat due to the presence of insect defoliators, including several species of chrysomelid beetles across several continents [20–22]. Of invasive pests in New Zealand, the most serious is the Eucalyptus Tortoise Beetle, *Paropsis charybdis* Stål 1860 (Coleoptera: Chrysomelidae: Chrysomelinae: Paropsini) [23, 24, 21]. *P*. *charybdis* has two generations per year in New Zealand, with the first generation adults emerging in August and September after overwintering within the leaf litter and under bark (spring generation) [23, 24] (Fig 1). There are four larval instars feeding on both expanding and adult leaves. Pupation of the spring generation occurs from November to December, and after approximately one month the second generation of beetles emerge (summer generation) [23, 24]. The summer generation appears the most damaging. Beetles are present until autumn, and thus a third generation has been proposed but not yet proven [25]. Although native to Australia, *P*. *charybdis* is now found virtually throughout New Zealand following its initial invasion to a localized region in 1916 [24]. The extensive damage caused by *P*. *charybdis* is likely the result of high female fecundity, wide host range, and a lack of natural enemies attacking the spring generation [26, 27].

Since the 1930s, classical biological control of *P*. *charybdis* in New Zealand has been attempted repeatedly through the importation and release of larval parasitoids (Tachinidae), egg parasitoids (Pteromalidae), and ladybird beetle predators (Coccinellidae) from Australia [28]. The majority of these agents failed to establish in New Zealand upon release [28]. The most successful biological control agent thus far was the pteromalid egg parasitoid, *Enoggera nassaui* (Girault, 1926) [28, 21]. This parasitoid was easily reared, had high rates of parasitism within the laboratory, and became established in a number of release locations that initially led to a substantial decline of *P*. *charybdis* populations. However, parasitism of the first generation of *P*. *charybdis* by *E*. *nassaui* was consistently low [26]. This was likely due to a phenological mismatch between parasitoid and host as the egg parasitoid was active too late in the spring (approximately 30 days following the appearance of *P*. *charybdis* eggs) [29].To rectify the phenological mismatch, wasps from a cooler region of Australia were introduced [26, 27]. Unfortunately, this agent has since been affected by an invasive obligate hyperparasitoid, *Baeoanusia albifuncile* Girault, (Hymenoptera: Encyrtidae), which reduced the success of the biological control program [30].

To date, biological control agents alone have not been able to control *P*. *charybdis* consistently. Insecticides are the only alternative for control [31, 32]. Alpha-cypermethrin, a broad-spectrum, synthetic pyrethroid can be used to control *P*. *charybdis* via aerial spraying. However, alpha-cypermethrin negatively impacts non-target fauna and thus, the Forest Stewardship Council (FSC), under which numerous *Eucalyptus* plantations in New Zealand are managed, has restricted the use of these chemicals [33, 32]. Research is now aimed at introducing a successful biological agent that is effective in the cooler climates of New Zealand and active during the first generation of *P*. *charybdis* [27, 34].

A new potential candidate is the solitary larval parasitoid, *Eadya paropsidis* Huddleston and Short 1978 (Hymenoptera: Braconidae: Euphorinae) [35, 36]. This wasp is univoltine and attacks the first generation of paropsine beetles feeding on *Eucalyptus* in Australia (Fig 1). *Eadya paropsidis* was described along with *E*. *falcata*, as the only two known species in the newly erected, Australian endemic genus [37]. The two species are widely separated geographically, with *E*. *paropsidis* known from the Australian Capital Territory, New South Wales, Victoria and Tasmania, and *E*. *falcata* known from Western Australia [37–39]. The biology of *E*. *falcata* is unknown, but *E*. *paropsidis* has been reared in the field from *Paropsis atomari*a Olivier 1807 (synonym *P*. *reticulata*) on mainland Australia [37, 39] and from *Paropsisterna bimaculata* (Olivier 1807) [38], *Paropsisterna agricola* (Chapuis 1987) [35], and *P*. *charybdis* in Tasmania (Allen, unpublished data). Although *Eadya* has been moved to Helconinae based on its placement in a one gene dataset [40], its biology and morphology and biology are consistent with its original placement in Euphorinae [41–43], including: attacking exposed chrysomelid beetles; forewing vein 2cu-a absent; forewing vein 3RS curved creating a small marginal cell; and metasomal tergum 1 petiolate [43]. In addition to these characters, species of *Eadya* can be identified by the presence of an inter-antennal carina and a closed second submarginal cell [43]. Rearing *Eadya* from field collections from a number of locations in Tasmania revealed two color morphs of the silk used to spin the wasp cocoon (Allen, unpublished data), suggesting the possibility of cryptic species of *Eadya*. However, due to a ten month obligate pupal diapause when much laboratory mortality happens, this species is frequently difficult to rear to an adult for morphological identification [36]. Hence using molecular phylogenetic approaches combined with host data from field collected paropsine beetle larvae, we set out to determine if *E*. *paropsidis* in Tasmania is: (1) one species or a group of cryptic species; (2) host-specific to *P*. *charybdis* and closely related Paropsini; and if so, (3) potentially suitable as an agent for biological control of *P*. *charybdis* in New Zealand. Wasps were collected from numerous localities across Tasmania over multiple years and reared to determine accurate associations with their paropsine beetle hosts. We utilized three molecular markers and morphology and present one of the most comprehensive datasets to investigate possible cryptic species and host specificity of a prospective classical biological control agent.

## Materials and methods

### Taxon sampling

**Fig 2:**
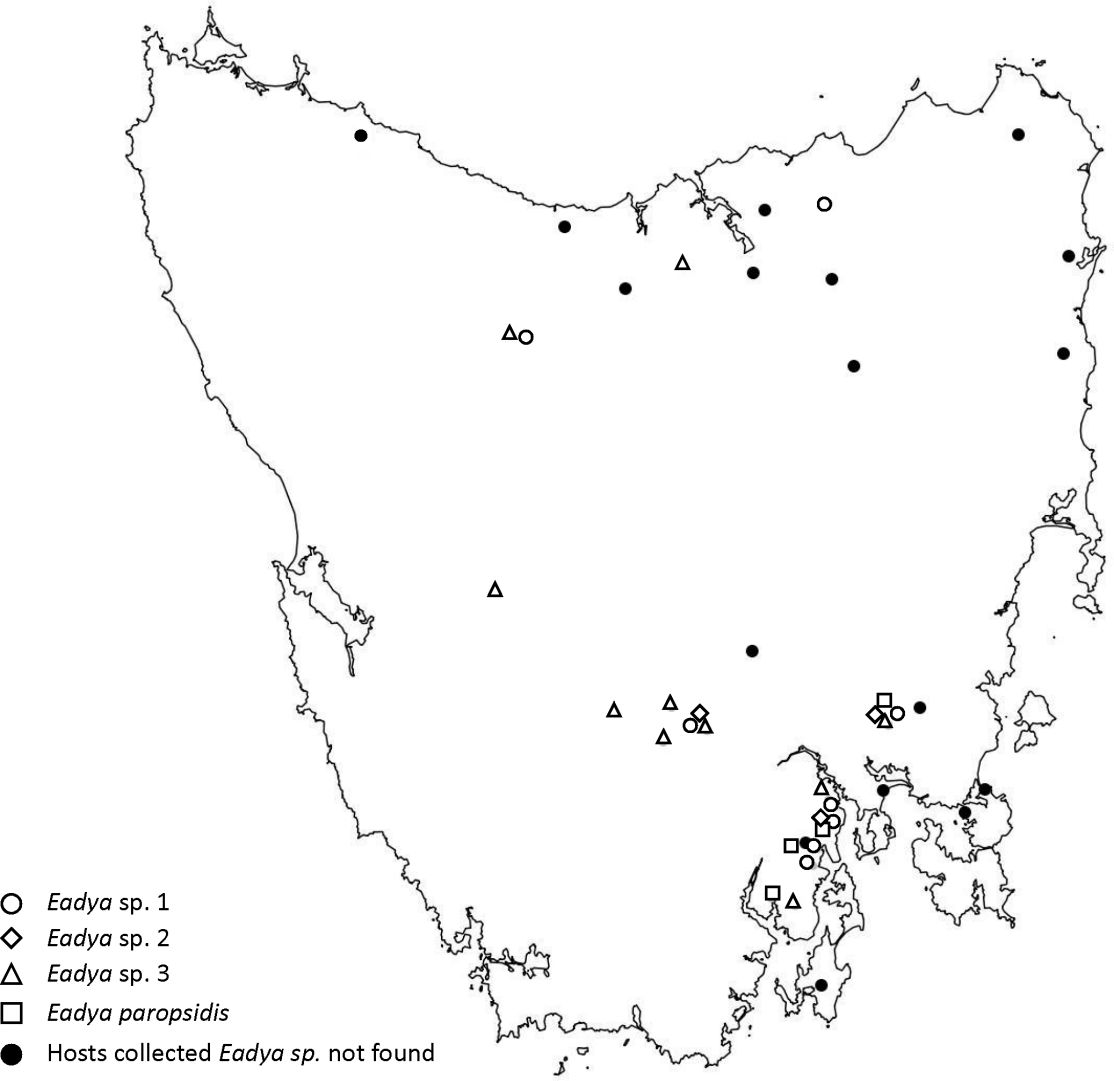
Map of collecting sites in Tasmania, Australia. All four *Eadya* species are shown, as well as collecting sites where no *Eadya* was found.

*Eadya* wasps and larval beetle hosts were collected from multiple field locations ranging from near sea level to sub-alpine (1000 m) in Tasmania, Australia across six years (2011–2016) from November to January (Fig 2). Specimens were collected by hand, sweep net, or malaise trap in the field. Wasps were reared to adulthood (n = 28), collected on the wing (n = 63) or dissected as larvae or pupae (n = 97) from collected paropsine beetle larvae (Table 1). Maps of beetle distributions by species are depicted in Supporting Information S1 Fig and the distribution of *Pst*. *selmani* Reid and de Little, 2013 across Tasmania can be found in Reid and de Little [44]. All Tasmanian collections were made from public land and roadsides not requiring permission with the exceptions of sampling and/or sentinel trials undertaken in plantations at Moina, Ellendale and Frankford, with permission obtained from Forestry Tasmania (Tim Wardlaw). Permission to collect at sites at Runnymede were obtained from Ifarm (Nick Martyn), and from 2016 onward from PF Olsen Australia (Robin Dickson). Comparative samples for beetles were obtained in New Zealand. Collections of *P*. *charybdis* were made with permission of the land manager at Poronui Station–Mr. Steve Smith, Westervelt Company, Taupo, New Zealand. New Zealand collections of *Pst*. *variicollis* were made under New Zealand Environmental Protection Authority permission for Scion to collect this species and breed it as a new organism in containment, approval number: NOC100191. Sampling at all locations did not involve endangered or protected species.

**Table 1:** Data collection table for *Eadya* from Tasmania, Australia.

Wasp Voucher #	Stage	Sex	Host	Location (Tasmania, Australia)	Year	Host Beetle Voucher #	Gene Amplified			Species Clade		Morpho- species	Phylo- species
							*CO1*	*Cytb*	*28S*	*COI*	*Cytb*		
BJS196	A	F	*P*. *charybdis*	Moina	2012		x	x	x	A	A	*E*. sp.1	*E*. sp.1
BJS214	L	?	*Pst*. *nobilitata*	Karanja	2013		x	x		A	A		*E*. sp.1
BJS215	L	?	*Pst*. *nobilitata*	Karanja	2013		x			A			*E*. sp.1
BJS216	L	?	*Pst*. variicollis*	Runnymede#2	2013	BJS509	x	x	x	A	A		*E*. sp.1
BJS217	L	?	*Pst*. variicollis*	Runnymede#2	2013	BJS510	x	x	x	A	A		*E*. sp.1
BJS218	L	?	*Pst*. variicollis*	Runnymede#2	2013		x	x	x	A	A		*E*. sp.1
BJS219	L	?	*Pst*. variicollis*	Runnymede#2	2013		x	x	x	A	A		*E*. sp.1
BJS220	L	?	*Pst*. variicollis*	Runnymede#2	2013	BJS512	x	x	x	A	A		*E*. sp.1
BJS221	L	?	*Pst*. variicollis*	Runnymede#2	2013		x	x	x	A	A		*E*. sp.1
BJS226	L	?	*P*. *charybdis*	Moina	2013		x			A			*E*. sp.1
BJS377	P	?	*Pst*. variicollis*	The Lea	2014		x	x	x	A	A		*E*. sp.1
BJS378	P	?	*Pst*. variicollis*	The Lea	2014		x	x	x	A	A		*E*. sp.1
BJS379	P	?	*Pst*. variicollis*	The Lea	2014		x	x		A	A		*E*. sp.1
BJS380	P	?	*Pst*. variicollis*	The Lea	2014	BJS465	x	x	x	A	A		*E*. sp.1
BJS381	P	?	*Pst*. variicollis*	The Lea	2014		x	x	x	A	A		*E*. sp.1
BJS382	P	?	*Pst*. variicollis*	The Lea	2014			x	x		A		*E*. sp.1
BJS383	P	?	*Pst*. variicollis*	The Lea	2014		x	x		A	A		*E*. sp.1
BJS384	P	?	*Pst*. variicollis*	The Lea	2014		x	x	x	A	A		*E*. sp.1
BJS385	L	?	*Pst*. variicollis*	The Lea	2014		x	x	x	A	A		*E*. sp.1
BJS386	L	?	*Pst*. variicollis*	The Lea	2014	BJS471	x	x		A	A		*E*. sp.1
BJS387	P	?	*Pst*. variicollis*	The Lea	2014		x	x	x	A	A		*E*. sp.1
BJS388	L	?	*Pst*. variicollis*	Runnymede#1	2014		x	x	x	A	A		*E*. sp.1
BJS403	L	?	*Pst*. *selmani*	Moina	2011		x	x	x	A	A		*E*. sp.1
BJS404	L	?	*Pst*. *selmani*	Moina	2011		x	x	x	A	A		*E*. sp.1
BJS405	L	?	*Pst*. *selmani*	Moina	2011		x		x	A			*E*. sp.1
BJS406	L	?	*Pst*. *selmani*	Moina	2011		x	x	x	A	A		*E*. sp.1
BJS407	L	?	*Pst*. *selmani*	Moina	2011		x	x	x	A	A		*E*. sp.1
BJS408	L	?	*Pst*. *selmani*	Moina	2011		x	x	x	A	A		*E*. sp.1
BJS409	L	?	*P*. *charybdis*	Moina	2011		x	x	x	A	A		*E*. sp.1
BJS501	A	M	*Pst*. variicollis*	The Lea	2014		x			A		*E*. sp.1	*E*. sp.1
BJS564	P	?	*Pst*. variicollis*	Runnymede#1	2015	BJS565	x	x	x	A	A		*E*. sp.1
BJS566	P	?	*Pst*. variicollis*	Runnymede#1	2015		x	x		A	A		*E*. sp.1
BJS199	A	F	*P*. *charybdis*	The Lea	2012	BJS200	x		x	B		*E*. sp.2	*E*. sp.2
BJS553	L	?	*P*. *aegrota elliotti*	Runnymede#2	2013	BJS559	x	x	x	B	n/a		*E*. sp.2
BJS204	A	F	*P*. *tasmanica*	Runnymede#1	2012		x	x		C	C	*E*. *paropsidis*	*E*. *paropsidis*
BJS205	A	F	*P*. *tasmanica*	Runnymede#1	2012		x	x	x	C	C	*E*. *paropsidis*	*E*. *paropsidis*
BJS206	P	?	*P*. *charybdis*	Runnymede#1	2012			x	x		C		*E*. *paropsidis*
BJS239	A	F	*P*. *tasmanica*	Runnymede#1	2012		x	x	x	C	C	*E*. *paropsidis*	*E*. *paropsidis*
BJS240	L	?	*P*. *tasmanica*	Runnymede#1	2013		x	x	x	C	C		*E*. *paropsidis*
BJS241	A	F	*P*. *tasmanica*	Runnymede#1	2012		x	x	x	C	C	*E*. *paropsidis*	*E*. *paropsidis*
BJS243	L	?	*P*. *tasmanica*	Runnymede#1	2012		x	x	x	C	C		*E*. *paropsidis*
BJS389	A	F	*P*. *tasmanica*	Runnymede#1	2012		x	x	x		C	*E*. *paropsidis*	*E*. *paropsidis*
BJS397	L	?	*P*. *tasmanica*	Runnymede#1	2013		x	x	x	C	C		*E*. *paropsidis*
BJS399	L	?	*P*. *tasmanica*	Runnymede#1	2013		x			C			*E*. *paropsidis*
BJS554	L	?	*P*. *tasmanica*	The Lea	2015		x	x	x	C	C		*E*. *paropsidis*
BJS562	P	?	*P*. *tasmanica*	Runnymede#1	2015			x			C		*E*. *paropsidis*
BJS175	L	?	*Pst*. *agricola*	Moina	2012		x	x	x	D	D		*E*. sp.3
BJS177	L	?	*Pst*. *agricola*	Moina	2012		x	x	x	D	D		*E*. sp.3
BJS179	L	?	*Pst*. *agricola*	Moina	2012		x	x	x	D	D		*E*. sp.3
BJS180	L	?	*Pst*. *agricola*	Moina	2012		x	x	x	D	D		*E*. sp.3
BJS182	L	?	*Pst*. *agricola*	Moina	2012		x	x	x	D	D		*E*. sp.3
BJS183	L	?	*Pst*. *agricola*	Moina	2012		x	x	x	D	D		*E*. sp.3
BJS184	L	?	*P*. *charybdis*	Ellendale	2012	BJS503	x	x	x	D	D		*E*. sp.3
BJS186	A	F	*P*. *charybdis*	Ellendale	2012	BJS504	x	x	x	D	D	*E*. sp.3	*E*. sp.3
BJS188	L	?	*Pst*. *agricola*	Moina	2012		x	x		D	D		*E*. sp.3
BJS189	L	?	*Pst*. *agricola*	Moina	2012		x	x	x	D	D		*E*. sp.3
BJS191	A	M	*Pst*. *agricola*	Moina	2012		x	x		D	D	*E*. sp.3	*E*. sp.3
BJS192	L	?	*P*. *charybdis*	Ellendale	2012		x	x		D	D		*E*. sp.3
BJS194	L	?	*Pst*. *agricola*	Ellendale	2012		x	x	x	D	D		*E*. sp.3
BJS202	A	F	unknown	Moina	2012		x	x	x	D	D	*E*. sp.3	*E*. sp.3
BJS203	A	M	unknown	Moina	2012		x	x	x	D	D	*E*. sp.3	*E*. sp.3
BJS213	L	?	*Pst*. *nobilitata*	Karanja	2013		x	x	x	D	D		*E*. sp.3
BJS223	L	?	*P*. *charybdis*	Ellendale	2013	BJS515	x	x	x	D	D		*E*. sp.3
BJS224	L	?	*P*. *charybdis*	Ellendale	2013	BJS516	x	x		D	D		*E*. sp.3
BJS225	L	?	*P*. *charybdis*	Ellendale	2013	BJS517	x	x	x	D	D		*E*. sp.3
BJS227	L	?	*Pst*. *agricola*	Moina	2013		x	x		D	D		*E*. sp.3
BJS228	L	?	*Pst*. *agricola*	Moina	2013	BJS520	x	x	x	D	D		*E*. sp.3
BJS229	L	?	*Pst*. *agricola*	Moina	2013	BJS521	x	x	x	D	D		*E*. sp.3
BJS230	L	?	*Pst*. *agricola*	Moina	2013	BJS522	x	x	x	D	D		*E*. sp.3
BJS231	L	?	*Pst*. *agricola*	Moina	2013		x	x	x	D	D		*E*. sp.3
BJS232	L	?	*Pst*. *agricola*	Moina	2013	BJS524	x		x	D			*E*. sp.3
BJS233	L	?	*Pst*. *agricola*	Runnymede#2	2013			x			D		*E*. sp.3
BJS234	L	?	*Pst*. *agricola*	Runnymede#2	2013	BJS526	x	x	x	D	D		*E*. sp.3
BJS235	L	?	*Pst*. *agricola*	Runnymede#2	2013		x	x	x	D	D		*E*. sp.3
BJS236	L	?	*Pst*. *agricola*	Runnymede#2	2013	BJS527	x	x	x	D	D		*E*. sp.3
BJS237	L	?	*Pst*. *agricola*	Runnymede#2	2013	BJS528	x	x	x	D	D		*E*. sp.3
BJS238	L	?	*Pst*. *agricola*	Runnymede#2	2013	BJS529	x	x	x	D	D		*E*. sp.3
BJS245	A	F	unknown	Moina	2013		x	x	x	D	D	*E*. sp.3	*E*. sp.3
BJS246	A	F	unknown	Moina	2013		x	x	x	D	D	*E*. sp.3	*E*. sp.3
BJS247	A	F	unknown	Moina	2013		x	x	x	D	D	*E*. sp.3	*E*. sp.3
BJS248	A	F	unknown	Moina	2013		x	x	x	D	D	*E*. sp.3	*E*. sp.3
BJS249	A	F	unknown	Moina	2013		x	x	x	D	D	*E*. sp.3	*E*. sp.3
BJS251	A	F	unknown	Moina	2013		x		x	D		*E*. sp.3	*E*. sp.3
BJS252	A	F	unknown	Moina	2013		x		x	D		*E*. sp.3	*E*. sp.3
BJS250	A	F	unknown	Moina	2013		x	x	x	D	D	*E*. sp.3	*E*. sp.3
BJS253	A	F	unknown	Moina	2013		x	x	x	D	D	*E*. sp.3	*E*. sp.3
BJS254	A	F	unknown	Moina	2013		x	x	x	D	D	*E*. sp.3	*E*. sp.3
BJS255	A	F	unknown	Moina	2013		x	x	x	D	D	*E*. sp.3	*E*. sp.3
BJS256	A	M	unknown	Moina	2013		x	x	x	D	D	*E*. sp.3	*E*. sp.3
BJS257	A	M	unknown	Moina	2013		x	x	x	D	D	*E*. sp.3	*E*. sp.3
BJS258	A	M	unknown	Moina	2013		x	x	x	D	D	*E*. sp.3	*E*. sp.3
BJS259	A	M	unknown	Moina	2013		x	x	x	D	D	*E*. sp.3	*E*. sp.3
BJS260	A	M	unknown	Moina	2013		x	x	x	D	D	*E*. sp.3	*E*. sp.3
BJS261	A	M	unknown	Moina	2013		x	x	x	D	D	*E*. sp.3	*E*. sp.3
BJS262	A	M	unknown	Moina	2013		x	x	x	D	D	*E*. sp.3	*E*. sp.3
BJS263	A	M	unknown	Moina	2013		x	x	x	D	D	*E*. sp.3	*E*. sp.3
BJS264	A	M	unknown	Moina	2013		x	x	x	D	D	*E*. sp.3	*E*. sp.3
BJS265	A	M	unknown	Moina	2013		x	x	x	D	D	*E*. sp.3	*E*. sp.3
BJS266	A	M	unknown	Moina	2013		x	x	x	D	D	*E*. sp.3	*E*. sp.3
BJS267	A	M	unknown	Moina	2013		x		x	D		*E*. sp.3	*E*. sp.3
BJS268	A	M	unknown	Moina	2013		x		x	D		*E*. sp.3	*E*. sp.3
BJS269	A	M	unknown	Moina	2013		x	x	x	D	D	*E*. sp.3	*E*. sp.3
BJS287	A	M	unknown	Moina	2014		x	x		D	D	*E*. sp.3	*E*. sp.3
BJS288	A	M	unknown	Moina	2014		x	x	x	D	D	*E*. sp.3	*E*. sp.3
BJS289	A	M	unknown	Moina	2014		x			D		*E*. sp.3	*E*. sp.3
BJS290	A	M	unknown	Moina	2014		x	x	x	D	D	*E*. sp.3	*E*. sp.3
BJS291	A	M	unknown	Moina	2014		x	x	x	D	D	*E*. sp.3	*E*. sp.3
BJS292	A	F	unknown	Moina	2014		x	x		D	D	*E*. sp.3	*E*. sp.3
BJS293	A	M	unknown	Runnymede#1	2014			x	x		D	*E*. sp.3	*E*. sp.3
BJS294	A	M	unknown	Runnymede#1	2014		x	x		D	D	*E*. sp.3	*E*. sp.3
BJS295	A	M	unknown	Runnymede#1	2014		x	x	x	D	D	*E*. sp.3	*E*. sp.3
BJS296	A	M	unknown	Runnymede#1	2014		x	x	x	D	D	*E*. sp.3	*E*. sp.3
BJS297	A	M	unknown	Runnymede#1	2014		x	x	x	D	D	*E*. sp.3	*E*. sp.3
BJS298	A	M	unknown	Runnymede#1	2014		x	x	x	D	D	*E*. sp.3	*E*. sp.3
BJS299	A	M	unknown	Runnymede#1	2014		x	x	x	D	D	*E*. sp.3	*E*. sp.3
BJS300	A	M	unknown	Runnymede#1	2014		x	x	x	D	D	*E*. sp.3	*E*. sp.3
BJS301	A	M	unknown	Runnymede#1	2014		x	x	x	D	D	*E*. sp.3	*E*. sp.3
BJS302	A	F	unknown	Runnymede#1	2014		x	x	x	D	D	*E*. sp.3	*E*. sp.3
BJS303	A	F	unknown	Runnymede#1	2014			x	x		D	*E*. sp.3	*E*. sp.3
BJS304	A	F	unknown	Runnymede#1	2014		x	x	x	D	D	*E*. sp.3	*E*. sp.3
BJS305	A	F	unknown	Runnymede#1	2014		x	x	x	D	D	*E*. sp.3	*E*. sp.3
BJS306	A	F	unknown	Runnymede#1	2014			x	x		D	*E*. sp.3	*E*. sp.3
BJS307	A	M	unknown	Runnymede#1	2014		x	x	x	D	D	*E*. sp.3	*E*. sp.3
BJS308	A	M	unknown	Runnymede#1	2014		x	x	x	D	D	*E*. sp.3	*E*. sp.3
BJS309	A	M	unknown	Runnymede#1	2014		x	x	x	D	D	*E*. sp.3	*E*. sp.3
BJS310	A	M	unknown	Runnymede#1	2014		x	x	x	D	D	*E*. sp.3	*E*. sp.3
BJS312	A	F	unknown	Runnymede#1	2014		x		x	D		*E*. sp.3	*E*. sp.3
BJS313	A	F	unknown	Runnymede#1	2014		x		x	D		*E*. sp.3	*E*. sp.3
BJS314	A	F	unknown	Runnymede#1	2014		x		x	D		*E*. sp.3	*E*. sp.3
BJS315	A	F	unknown	Ellendale	2014		x	x	x	D	D	*E*. sp.3	*E*. sp.3
BJS316	A	F	unknown	Ellendale	2014		x	x	x	D	D	*E*. sp.3	*E*. sp.3
BJS317	A	F	unknown	Ellendale	2014		x	x	x	D	D	*E*. sp.3	*E*. sp.3
BJS318	A	F	unknown	Ellendale	2014		x	x	x	D	D	*E*. sp.3	*E*. sp.3
BJS319	A	M	unknown	Moina	2014		x	x	x	D	D	*E*. sp.3	*E*. sp.3
BJS320	A	M	unknown	Moina	2014		x	x	x	D	D	*E*. sp.3	*E*. sp.3
BJS321	A	M	unknown	Moina	2014		x	x	x	D	D	*E*. sp.3	*E*. sp.3
BJS322	A	M	unknown	Moina	2014		x	x	x	D	D	*E*. sp.3	*E*. sp.3
BJS323	A	M	unknown	Moina	2014		x	x	x	D	D	*E*. sp.3	*E*. sp.3
BJS324	A	F	*Pst*. *agricola*	Ellendale	2014		x	x	x	D	D	*E*. sp.3	*E*. sp.3
BJS325	A	F	*Pst*. *agricola*	Ellendale	2014		x	x	x	D	D	*E*. sp.3	*E*. sp.3
BJS326	A	F	*Pst*. *agricola*	Ellendale	2014		x	x	x	D	D	*E*. sp.3	*E*. sp.3
BJS327	A	F	*Pst*. *agricola*	Ellendale	2014	BJS414	x	x	x	D	D	*E*. sp.3	*E*. sp.3
BJS328	A	F	*Pst*. *agricola*	Ellendale	2014	BJS415	x	x	x	D	D	*E*. sp.3	*E*. sp.3
BJS329	A	F	*Pst*. *agricola*	Ellendale	2014	BJS416	x	x	x	D	D	*E*. sp.3	*E*. sp.3
BJS330	A	F	*Pst*. *agricola*	Ellendale	2014		x	x	x	D	D	*E*. sp.3	*E*. sp.3
BJS331	A	M	*Pst*. *agricola*	Ellendale	2014	BJS418	x	x	x	D	D	*E*. sp.3	*E*. sp.3
BJS332	A	M	*Pst*. *agricola*	Ellendale	2014		x	x	x	D	D	*E*. sp.3	*E*. sp.3
BJS333	A	M	*Pst*. *agricola*	Ellendale	2014	BJS420	x	x	x	D	D	*E*. sp.3	*E*. sp.3
BJS334	A	M	*Pst*. *agricola*	Ellendale	2014		x	x	x	D	D	*E*. sp.3	*E*. sp.3
BJS335	A	M	*Pst*. *agricola*	Moina	2014		x	x	x	D	D	*E*. sp.3	*E*. sp.3
BJS336	A	F	*Pst*. *agricola*	Moina	2014	BJS423	x	x	x	D	D	*E*. sp.3	*E*. sp.3
BJS337	A	F	*Pst*. *agricola*	Runnymede#1	2014	BJS424	x	x	x	D	D	*E*. sp.3	*E*. sp.3
BJS338	A	F	*Pst*. *agricola*	Runnymede#1	2014	BJS425	x	x	x	D	D	*E*. sp.3	*E*. sp.3
BJS339	A	F	*Pst*. *agricola*	Runnymede#1	2014	BJS426	x	x	x	D	D	*E*. sp.3	*E*. sp.3
BJS341	L	?	*Pst*. *agricola*	Runnymede#1	2014	BJS428	x	x	x	D	D		*E*. sp.3
BJS342	L	?	*Pst*. *agricola*	Runnymede#1	2014			x	x		D		*E*. sp.3
BJS343	L	?	*Pst*. *agricola*	Runnymede#1	2014			x	x		D		*E*. sp.3
BJS344	L	?	*Pst*. *agricola*	Runnymede#1	2014	BJS431	x	x	x	D	D		*E*. sp.3
BJS345	L	?	*Pst*. *agricola*	Runnymede#1	2014	BJS432	x	x	x	D	D		*E*. sp.3
BJS346	L	?	*Pst*. *agricola*	Runnymede#1	2014		x	x	x	D	D		*E*. sp.3
BJS347	L	?	*Pst*. *agricola*	Runnymede#1	2014	BJS434	x	x	x	D	D		*E*. sp.3
BJS348	L	?	*Pst*. *agricola*	Runnymede#1	2014	BJS435	x	x	x	D	D		*E*. sp.3
BJS349	L	?	*Pst*. *agricola*	Runnymede#1	2014	BJS436	x	x	x	D	D		*E*. sp.3
BJS350	L	?	*Pst*. *agricola*	Ellendale	2014	BJS437	x	x	x	D	D		*E*. sp.3
BJS351	L	?	*Pst*. *agricola*	Ellendale	2014	BJS438	x	x	x	D	D		*E*. sp.3
BJS352	L	?	*Pst*. *agricola*	Ellendale	2014		x	x	x	D	D		*E*. sp.3
BJS353	L	?	*Pst*. *agricola*	Ellendale	2014		x	x	x	D	D		*E*. sp.3
BJS354	L	?	*Pst*. *agricola*	Ellendale	2014	BJS441	x	x	x	D	D		*E*. sp.3
BJS355	L	?	*Pst*. *agricola*	Ellendale	2014	BJS442	x	x		D	D		*E*. sp.3
BJS359	L	?	*Pst*. *agricola*	Moina	2014		x	x	x	D	D		*E*. sp.3
BJS361	L	?	*Pst*. *agricola*	Moina	2014	BJS447	x	x	x	D	D		*E*. sp.3
BJS362	L	?	*Pst*. *agricola*	Moina	2014			x	x		D		*E*. sp.3
BJS363	L	?	*Pst*. *agricola*	Moina	2014	BJS449	x	x	x	D	D		*E*. sp.3
BJS364	L	?	*Pst*. *agricola*	Moina	2014		x	x	x	D	D		*E*. sp.3
BJS366	L	?	*Pst*. *agricola*	Moina	2014	BJS451	x	x	x	D	D		*E*. sp.3
BJS367	L	?	*Pst*. *agricola*	Moina	2014	BJS452	x	x	x	D	D		*E*. sp.3
BJS368	L	?	*Pst*. *agricola*	Moina	2014	BJS453	x	x		D	D		*E*. sp.3
BJS369	P	?	*Pst*. *agricola*	Moina	2014	BJS454	x	x	x	D	D		*E*. sp.3
BJS370	P	?	*Pst*. *agricola*	Moina	2014		x	x	x	D	D		*E*. sp.3
BJS371	L	?	*Pst*. *agricola*	Runnymede#1	2015		x		x	D			*E*. sp.3
BJS372	L	?	*P*. *charybdis*	Runnymede#1	2014	BJS457	x	x	x	D	D		*E*. sp.3
BJS373	L	?	*Pst*. *bimaculata*	Ellendale	2014				x				*E*. sp.3
BJS374	A	M	*Pst*. *agricola*	Moina	2014	BJS459	x	x	x	D	D	*E*. sp.3	*E*. sp.3
BJS376	P	?	*Pst*. *bimaculata*	Moina	2014	BJS461	x	x		D	D		*E*. sp.3
BJS391	A	F	*Pst*. *agricola*	Moina	2013		x	x		D	D	*E*. sp.3	*E*. sp.3
BJS393	L	?	*Pst*. *agricola*	Ellendale	2012	BJS477	x	x		D	D		*E*. sp.3
BJS394	L	?	*Pst*. *agricola*	Ellendale	2012			x			D		*E*. sp.3
BJS410	L	?	*Pst*. *agricola*	Moina	2012		x	x		D	D		*E*. sp.3

Voucher numbers are referenced in Genbank Accession Numbers. Stage: A = Adult, L = Larva. Sex is only known for adult wasps. For host, *P*. = *Paropsis*, *Pst*. = *Paropsisterna*; *Pst*. variicollis* refers to the *Pst*. *variicollis* complex whose taxonomic status across southern Australia is unresolved and hence was undeterminable to an exact phylospecies. If the wasp was reared from a beetle that was extracted for DNA, the beetle voucher number is listed. Successful amplification of genes is listed with an “x” under the appropriate gene. For species clades and phylospecies, refer to the phylogenetic analyses (see results).

Additional *Eadya* specimens were obtained through sentinel larval trials. These trials involved placing laboratory-reared, parasitoid free, 2^nd^ instar paropsine larvae on *E*. *nitens* branches in the field to assess levels of parasitism by species of *Eadya*. On each *E*. *nitens* tree, sentinel larvae were placed on foliage of a branch of approximately 1 cm diameter that was tied down firmly to a stake in the ground to prevent contact with other branches, and hence loss of sentinel larvae to neighboring branches. The stake and the branch leading to the main stem were smothered in Tanglefoot™ (The Scotts Company, Ohio, USA) to reduce predation and larval wandering. Branch foliage was then clipped back to approximately 0.33 m^2^. All insects and spiders that were located on that foliage were carefully removed. When confident that the foliage was free of arthropods, laboratory-reared beetle larvae were released onto each branch. Larvae were left for 72 hours before those remaining were carefully removed from each branch, into separate plastic aerated containers, one for each replicate, and returned to the laboratory for rearing to pupation or wasp emergence within a Contherm^TM^ chamber set at 20 ± 1°C and 16:8 L:D cycle. Emerged parasitoids were preserved in ethanol for molecular analysis.

Three different paropsine beetles were reared in the laboratory for the sentinel trials: *P*. *charybdis*, *Pst*. *agricola*, and *Pst*. *selmani*. *Paropsis charybdis* larvae were obtained from colonies initiated each season from adults collected from Hobart, Tasmania off *Eucalyptus ovata* and *E*. *viminalis*. Pairs were maintained in cages at the University of Tasmania with *E*. *viminalis* branches at room temperature. Larvae of *Pst*. *agricola* and *Pst*. *selmani* were obtained as eggs laid on juvenile foliage of *E*. *nitens* from Moina (41°32'27"S 146°04'38"E), Northern Tasmania and maintained in the laboratory on cut juvenile leaves of *E*. *nitens*. A preliminary sentinel trial was conducted in an *E*. *nitens* plantation in Moina in December 2011 to establish appropriate methodology. For each replicate (tree), 25 larvae were placed per branch, with 6 replicates of *Pst*. *agricola* and *Pst*. *selmani*, and 4 replicates *of P*. *charybdis*. The sentinel trials were repeated between the 5^th^ and 18^th^ of December 2012 using just *P*. *charybdis* (n = 767) and *Pst*. *agricola* (n = 394) with higher numbers of larvae per tree (either 50 or 100) at the following sites: Ellendale (42°38'07.24"S 146°45'04.24"E) (4 replicates per species), Moina (3 replicates per species), Runnymede (42°38'08.9"S 147°33'57.9"E) (3 replicates of *P*. *charybdis*), Mount Nelson (45°55' 42"S 147° 18’25"E) (4 replicates of *P*. *charybdis*), and The Lea (45°56'43"S 147°18'50"E) (2 replicates of *P*. *charybdis*), with the latter two sites being native vegetation rather than plantation sites.

Wherever possible, since paropsine beetles typically lay eggs in batches, reared *Eadya* specimens for molecular determination were taken from differing host larval groupings to maximize the chance that each *Eadya* were from different mothers. Beetle hosts included eight species from two different genera (*Paropsis* (abbreviated *P*.) and *Paropsisterna* (abbreviated as *Pst*.): *Pst*. *agricola*, *Pst*. *bimaculata*, *Pst*. *nobilitata* (Erichson 1842), *Pst*. *selmani* (only recovered from sentinel trials), *Pst*. *variicollis* (Chapuis 1877), *P*. *aegrota* *elliotti* Selman, 1983, *P*. *charybdis*, and *P*. *tasmanica* (Tables 1 and 2). The taxonomic status of *Pst*. *variicollis* is not clear, particularly with respect to two other names in use, *Pst*. *obovata* (Chapuis, 1877) and *Pst*. *cloelia* (Stål, 1860) (Chris Reid, Australian Museum, personal communication). This binomial could be valid or it may be a synonym of *Pst*. *cloelia*, and thus we refer to this taxon as *Pst*. variicollis* for the remainder of the paper to prevent further confusion. Further, an urgent revision is needed due to the recent invasion of New Zealand of *Pst*. variicollis*. Adult beetle voucher specimens were also sampled to have an accurately identified reference library to compare with DNA extracted from putatively identified beetle larvae (Table 2). This is important for field collected hosts, as larval paropsine beetle identifications can be challenging. Finally, several specimens of *P*. *charybdis*, *Pst*. variicollis* and one specimen of *Pst*. *beata* (Newman 1842) collected from New Zealand were also sampled (Table 2). All wasp and beetle voucher specimens are maintained at the University of Central Florida Collection of Arthropods or the Australian National Insect Collection (Tables 1 and 2 and S1 Table).

**Table 2:** Data collection table for paropsine beetles.

Beetle Voucher #	Stage	Method (F = Field, S = Sentinel)	Identified as	Location	Year	Wasp Voucher #	Phylospecies
BJS200	L	F	*P*. *charybdis*	The Lea	2012	BJS199	*P*. *charybdis*
BJS201	L	S	*P*. *charybdis*	The Lea	2012	n/a	*P*. *charybdis*
BJS270	A	F	*P*. *charybdis*	Upper Hutt, NZL	2014	n/a	*P*. *charybdis*
BJS271	A	F	*P*. *charybdis*	Upper Hutt, NZL	2014	n/a	*P*. *charybdis*
BJS273	A	F	*P*. *charybdis*	Upper Hutt, NZL	2014	n/a	*P*. *charybdis*
BJS274	A	F	*P*. *charybdis*	Upper Hutt, NZL	2014	n/a	*P*. *charybdis*
BJS275	A	F	*P*. *charybdis*	Upper Hutt, NZL	2014	n/a	*P*. *charybdis*
BJS276	A	F	*P*. *charybdis*	Upper Hutt, NZL	2014	n/a	*P*. *charybdis*
BJS277	A	F	*P*. *charybdis*	Upper Hutt, NZL	2014	n/a	*P*. *charybdis*
BJS278	A	F	*P*. *charybdis*	Upper Hutt, NZL	2014	n/a	*P*. *charybdis*
BJS280	A	F	*P*. *charybdis*	Upper Hutt, NZL	2014	n/a	*P*. *charybdis*
BJS281	A	F	*P*. *charybdis*	Upper Hutt, NZL	2014	n/a	*P*. *charybdis*
BJS282	A	F	*P*. *charybdis*	Upper Hutt, NZL	2014	n/a	*P*. *charybdis*
BJS283	A	F	*P*. *charybdis*	Upper Hutt, NZL	2014	n/a	*P*. *charybdis*
BJS284	A	F	*P*. *charybdis*	Upper Hutt, NZL	2014	n/a	*P*. *charybdis*
BJS285	A	F	*P*. *charybdis*	Upper Hutt, NZL	2014	n/a	*P*. *charybdis*
BJS286	A	F	*P*. *charybdis*	Upper Hutt, NZL	2014	n/a	*P*. *charybdis*
BJS414	L	F	*Pst*. *agricola*	Ellendale	2014	BJS327	*Pst*. *agricola*
BJS415	L	F	*Pst*. *agricola*	Ellendale	2014	BJS328	*Pst*. *agricola*
BJS416	L	F	*Pst*. *agricola*	Ellendale	2014	BJS329	*Pst*. *agricola*
BJS418	L	F	*Pst*. *agricola*	Ellendale	2014	BJS331	*Pst*. *agricola*
BJS420	L	F	*Pst*. *agricola*	Ellendale	2014	BJS333	*Pst*. *agricola*
BJS423	L	F	*Pst*. *agricola*	Moina	2014	BJS336	*Pst*. *agricola*
BJS424	L	F	*Pst*. *agricola*	Runnymede#1	2014	BJS337	*Pst*. *agricola*
BJS425	L	F	*Pst*. *agricola*	Runnymede#1	2014	BJS338	*Pst*. *agricola*
BJS426	L	F	*Pst*. *agricola*	Runnymede#1	2014	BJS339	*Pst*. *agricola*
BJS428	L	F	*Pst*. *agricola*	Runnymede#1	2014	BJS341	*Pst*. *agricola*
BJS429	L	F	*Pst*. *agricola*	Runnymede#1	2014	n/a	*Pst*. *agricola*
BJS430	L	F	*Pst*. *agricola*	Runnymede#1	2014	n/a	*Pst*. *agricola*
BJS431	L	F	*Pst*. *agricola*	Runnymede#1	2014	BJS344	*Pst*. *agricola*
BJS432	L	F	*Pst*. *agricola*	Runnymede#1	2014	BJS345	*Pst*. *agricola*
BJS434	L	F	*Pst*. *agricola*	Runnymede#1	2014	BJS347	*Pst*. *agricola*
BJS435	L	F	*Pst*. *agricola*	Runnymede#1	2014	BJS348	*Pst*. *agricola*
BJS436	L	F	*Pst*. *agricola*	Runnymede#1	2014	BJS349	*Pst*. *agricola*
BJS437	L	F	*Pst*. *agricola*	Ellendale	2014	BJS350	*Pst*. *agricola*
BJS438	L	F	*Pst*. *agricola*	Ellendale	2014	BJS351	*Pst*. *agricola*
BJS441	L	F	*Pst*. *agricola*	Ellendale	2014	BJS354	*Pst*. *agricola*
BJS442	L	F	*Pst*. *agricola*	Ellendale	2014	BJS355	*Pst*. *agricola*
BJS443	L	F	*Pst*. *agricola*	Ellendale	2014	n/a	*Pst*. *agricola*
BJS447	L	F	*Pst*. *agricola*	Moina	2014	BJS361	*Pst*. *agricola*
BJS448	L	F	*Pst*. *agricola*	Moina	2014	n/a	*Pst*. *agricola*
BJS449	L	F	*Pst*. *agricola*	Moina	2014	BJS363	*Pst*. *agricola*
BJS451	L	F	*Pst*. *agricola*	Moina	2014	BJS366	*Pst*. *agricola*
BJS452	L	F	*Pst*. *agricola*	Moina	2014	BJS367	*Pst*. *agricola*
BJS453	L	F	*Pst*. *agricola*	Moina	2014	BJS368	*Pst*. *agricola*
BJS454	L	F	*Pst*. *agricola*	Moina	2014	BJS369	*Pst*. *agricola*
BJS457	L	F	*P*. *charybdis*	Runnymede#1	2014	BJS372	*P*. *charybdis*
BJS459	L	F	*Pst*. *agricola*	Moina	2014	BJS374	*Pst*. *agricola*
BJS460	L	F	*Pst*. *agricola*	Moina	2014	n/a	*Pst*. *agricola*
BJS461	L	F	*Pst*. *bimaculata*	Moina	2014	BJS376	*Pst*. *bimaculata*
BJS465	L	F	*Pst*. variicollis*	The Lea	2014	BJS380	*Pst*. variicollis*
BJS471	L	F	*Pst*. variicollis*	The Lea	2014	BJS386	*Pst*. variicollis*
BJS477	L	F	*Pst*. *agricola*	Ellendale	2012	BJS393	*Pst*. *agricola*
BJS503	L	S	*P*. *charybdis*	Ellendale	2012	BJS184	*P*. *charybdis*
BJS504	L	S	*P*. *charybdis*	Ellendale	2012	BJS186	*P*. *charybdis*
BJS506	L	S	*P*. *charybdis*	Runnymede#1	2012	n/a	*P*. *charybdis*
BJS509	L	F	*Pst*. variicollis*	Runnymede#2	2013	BJS216	*Pst*. variicollis*
BJS510	L	F	*Pst*. variicollis*	Runnymede#2	2013	BJS217	*Pst*. variicollis*
BJS512	L	F	*Pst*. variicollis*	Runnymede#2	2013	BJS220	*Pst*. variicollis*
BJS515	L	F	*P*. *charybdis*	Ellendale	2013	BJS223	*P*. *charybdis*
BJS516	L	F	*P*. *charybdis*	Ellendale	2013	BJS224	*P*. *charybdis*
BJS517	L	F	*P*. *charybdis*	Ellendale	2013	BJS225	*P*. *charybdis*
BJS518	L	F	*P*. *charybdis*	Ellendale	2013	n/a	*P*. *charybdis*
BJS520	L	F	*Pst*. *agricola*	Moina	2013	BJS228	*Pst*. *agricola*
BJS521	L	F	*Pst*. *agricola*	Moina	2013	BJS229	*Pst*. *agricola*
BJS522	L	F	*Pst*. *agricola*	Moina	2013	BJS230	*Pst*. *agricola*
BJS524	L	F	*Pst*. *agricola*	Moina	2013	BJS232	*Pst*. *agricola*
BJS526	L	F	*Pst*. *agricola*	Runnymede#2	2013	BJS234	*Pst*. *agricola*
BJS527	L	F	*Pst*. *agricola*	Runnymede#2	2013	BJS236	*Pst*. *agricola*
BJS528	L	F	*Pst*. *agricola*	Runnymede#2	2013	BJS237	*Pst*. *agricola*
BJS529	L	F	*Pst*. *agricola*	Runnymede#2	2013	BJS238	*Pst*. *agricola*
BJS531	A	F	*P*. *charybdis*	Olinda Grove	2014	n/a	*P*. *charybdis*
BJS532	A	F	*Pst*. *decolorata*	Olinda Grove	2014	n/a	*Pst*. *decolorata*
BJS533	A	F	*P*. *tasmanica*	Olinda Grove	2014	n/a	*P*. *tasmanica*
BJS535	A	F	*P*. *charybdis*	Runnymede#1	2014	n/a	*P*. *charybdis*
BJS537	A	F	*Pst*. *agricola*	Runnymede#1	2014	n/a	*Pst*. *agricola*
BJS539	A	F	*Pst*. *bimaculata*	Ellendale	2014	n/a	*Pst*. *bimaculata*
BJS543	A	F	*P*. *aegrota elliotti*	Moina	2014	n/a	*P*. *aegrota elliotti*
BJS545	A	F	*Pst*. variicollis*	Seven Miles Beach	2014	n/a	*Pst*. variicollis*
BJS547	A	F	*Pst*. *decolorata*	Runnymede#1	2014	n/a	*Pst*. *decolorata*
BJS557	L	F	*P*. *tasmanica*	The Lea	2015	n/a	*P*. *tasmanica*
BJS558	L	F	*P*. *aegrota elliotti*	Runnymede#2	2013	n/a	*Pst*. variicollis*
BJS559	L	F	*P*. *aegrota elliotti*	Runnymede#2	2013	BJS553	*P*. *aegrota elliotti*
BJS561	L	F	*P*. *tasmanica*	Runnymede#2	2015	n/a	*P*. *tasmanica*
BJS565	L	F	*Pst*. variicollis*	Runnymede#1	2015	BJS564	*Pst*. variicollis*
BJS568	A	F	*Pst*. variicollis*	Hawkes Bay, NZL	2016	n/a	*Pst*. variicollis*
BJS569	A	F	*Pst*. variicollis*	Hawkes Bay, NZL	2016	n/a	*Pst*. variicollis*
BJS570	A	F	*Pst*. variicollis*	Hawkes Bay, NZL	2016	n/a	*Pst*. variicollis*
BJS572	A	F	*Pst*. variicollis*	Hawkes Bay, NZL	2016	n/a	*Pst*. variicollis*
BJS573	A	F	*Pst*. variicollis*	Hawkes Bay, NZL	2016	n/a	*Pst*. variicollis*
BJS574	A	F	*Pst*. variicollis*	Hawkes Bay, NZL	2016	n/a	*Pst*. variicollis*
BJS575	A	F	*Pst*. variicollis*	Hawkes Bay, NZL	2016	n/a	*Pst*. variicollis*
T12-4654A	A	F	*Pst*. *beata*	Upper Hutt, NZL	2012	n/a	*Pst*. *beata*
T16-01003B	A	F	*Pst*. variicollis*	Napier, NZL	2016	n/a	*Pst*. variicollis*

Voucher numbers are referenced in Genbank Accession Numbers. All locations are in Tasmania, except where noted. Stage: A = Adult, L = Larva. If the beetle was host to a parasitoid that was extracted for DNA, the wasp voucher number is listed. The identified species was the original identification before analysis, and phylospecies based on the phylogenetic analysis of *COI*. *P*. = *Paropsis*, *Pst*. = *Paropsisterna*; *Pst*. variicollis* refers to the *Pst*. *variicollis* complex whose taxonomic status across southern Australia is unresolved (see text)

### Genetic sampling

A total of 188 wasps and 94 beetles were extracted for DNA and molecular analysis. Three gene regions were amplified, including two mitochondrial genes (Cytochrome oxidase I [*COI*] and Cytochrome b [*Cytb*]) and one nuclear gene (28S rDNA regions D1-D3 [*28S*]). *COI* has long been the standard for species delimitation in insects [45, 16, 46, 18, 47] including Braconidae [48, 49, 17, 19]. *Cytb* is generally more conserved but can help provide an independent test to prevent overestimations of species [50]. Additionally, *28S* has several variable regions (i.e., regions of ambiguous alignment) [51, 52] that could potentially provide useful characters for species delimitation and thus was selected for amplification.

DNA was extracted and genes amplified from wasps (Table 1) and a subset of their beetle hosts (Table 2). Genomic DNA extraction of the wasps and beetle hosts was done using the DNEasy Mini Kit (Qiagen). The metasoma was separated from the adult and dissected pupal wasps to increase DNA concentration and ensure a voucher specimen was available post-extraction for morphological examination. Larval wasps that had emerged from their host (prior to pupation) were ground with a sterilized pestle prior to extraction. Similarly, the associated beetle larvae from which the wasp emerged was also pulverized prior to extraction to ensure adequate DNA recovery as most beetle hosts were in poor condition after parasitization. DNA was also extracted from beetles collected as adults and vouchers retained and only *COI* was amplified as a tool to provide barcode confirmations on larval identifications. All PCR reactions were performed using 0.2–1 μg DNA extract, 1 X Standard Taq Buffer (New England Biolabs (NEB), U.S.A.) (10 mm Tris-HCl, 50 mm KCl, 1.5 mm MgCl2), 200 μm dNTP (NEB), 4 mm MgSO, 400 nm of each primer, 1 unit of Taq DNA polymerase (NEB) and purified water to a final volume of 25 μL. All primers and associated thermal cycling conditions are listed in S2 Table. Reaction products were cleaned with Agencourt CleanSEQ magnetic beads and sequenced in both directions using the BigDye Terminator Cycle Sequencing Kit (Applied Biosystems, U.S.A.) and the Applied Biosystems 3730xl DNA Analyzer at the University of Kentucky, Advanced Genetic Technologies Center (UK-AGTC). Contigs were assembled and sequences edited for quality using Geneious v. 8.1.8 [53]. Sequences were deposited in GenBank under accession numbers KX989891-KX990220, KY031346-KY031518 and MH107809-MH107817 for *Eadya* and MH237732-MH237825 for beetles.

### Phylogenetic analyses

Gene alignments were completed for *COI* and *Cytb* by hand using the reading frame as a guide with Bioedit v.7.1.3 [54]. There were no indels present in either gene and thus the alignments were unambiguous. A modified [52] secondary structure model [51] was used to align *28S*. Regions of ambiguous alignment (RAAs), and regions of expansion and contraction (RECs) were not excluded from the data set as we assumed most informative characters for this gene would be contained within these regions that can be hyper variable across genera. For each gene, the best fitting model of DNA sequence evolution for nucleotide analyses were determined using jModelTest v.0.1.1 [55] under the Bayesian Information Criterion (BIC). The model with the lowest calculated BIC score was considered the best-fitting model for each gene. Depending on the gene, either one or two species from other braconid subfamilies were used as outgroups *Afrocampsis* sp. (Acampsohelconinae) and *Eumacrocentrus americanus* (Cresson 1873) (Helconinae) to ensure the ingroup was monophyletic.

For individual and concatenated data sets, Bayesian inference with two independent runs each with four chains and default priors was run in MrBayes v.3.2.6 [56]. An independent molecular model was applied to each partition in the concatenated data set (partitioned by gene) and different parameters of the model were unlinked to allow each partition to have its own set of estimations for parameters. The rate parameter was set to vary across different partitions to incorporate rate heterogeneity across partitions. Data sets for all gene alignments were deposited in Figshare (https://figshare.com/): 10.6084/m9.figshare.6149219.

All analyses were performed for 5,000,000 generations sampling every 1000^th^ generation and the results from the two independent runs were summarized in a majority rule consensus tree after discarding the initial 25% of the trees for burn-in. Stationarity and appropriate mixing of the two independent runs were determined when the average standard deviation of split frequencies approached 0.01, the Potential Scale Reduction Factor (PSRF) for each parameter of the model was close to 1, and the overlay plots of both runs showed the number of generations versus the log probability of the data were heteroscedastic. Average intraclade and interclade genetic distances were calculated using Kimura’s two-parameter model [57] using MEGA v.7.0.14 [58].

### Morphological examination

We examined all adult specimens of *Eadya* collected for the purposes of this study (Table 1, adults), plus additional specimens collected in malaise traps, the paratype of *E*. *paropsidis*, the holotype and paratype of *E*. *falcata*, and some museum specimens (see additional material examined in S1 Table). We initially sorted specimens into morphotypes and observed four distinct morphospecies. One perfectly matched the description and paratype of *E*. *paropsidis*. Based on examination of all material, *Eadya* sp. 1 and sp. 2 were morphologically distinct with observable differences in several morphological characters from *E*. *paropsidis* or *E*. *falcata*. For example, *Eadya* sp. 1 and 2 do not possess a transverse carinae on the propodeum as in *E*. *paropsidis*, and have more impressed notauli than *E*. *falcata*. Further, *Eadya* sp. 1 lack median tubercles on the clypeus. However, the fourth morphospecies was very similar to *E*. *paropsidis* but was distinctly smaller in size. Parasitoids can vary in size due to the size of their host and nutritional factors during larval development. However, in their original description of *Eadya*, Huddleston and Short (37) noted variation across *E*. *paropsidis*, particularly a series of eight specimens that were smaller in size and had a less concave occiput. Although they chose not to describe these variants as a new species, we chose to separate the smaller specimens (as *Eadya* sp. 3) to test whether or not it was indeed a distinct species. Although we discuss the molecular results in context with the morphological examinations of morphospecies, descriptions of the new species are fully described in a separate paper as *Eadya annleckiae* Ridenbaugh 2018 (sp. 1); *Eadya spitzer* Ridenbaugh 2018 (sp. 2), and *Eadya daenerys* Ridenbaugh 2018 (sp. 3) [43].

## Results

### Parasitism rates of paropsine beetles

A total of 2924 field collected paropsine beetle larvae across 10 beetle species, comprising over 135 independent collections (groups of larvae from same egg batch) were reared over six years (Table 3). Four beetle species had substantially higher (>18%) parasitism rates: *P*. *tasmanica*, *P*. variicollis*, *P*. *charybdis* and *Pst*. *agricola*, whereas no *Eadya* were reared from three beetle species. For the sentinel trials, the number of larvae recovered (n = 616) and the percent parasitized by *Eadya* or unidentified Tachinidae is presented in Table 4 for the preliminary trial at Moina in 2011 and the more substantial trials at five locations in 2012. The lack of parasitism of *Pst*. *agricola* by *Eadya* at Moina in 2011 was unexpected, but the timing of the trial was very late in the flight season for the species of *Eadya* that parasitizes this host. In the 2012 trials, species of *Eadya* parasitized beetle larvae at four of the five sites (Table 4). At two of the plantation sites in 2012, there were high levels of parasitism by tachinid flies.

**Table 3:** Summary of field collected beetle larvae collected across various locations and years across Tasmania and the overall parasitism rate by *Eadya*.

Beetle Species	No. Collected	No. Independent Collections	No. Locations	No. Years	Parasitism Rate (%)
*Paropsis aegrota elliotti* Selman, 1983	89	17	11	3	2.2
*Paropsis charybdis* Stål, 1860	65	13	9	4	18.0
*Paropsis delittlei* Selman 1983	92	6	3	2	0
*Paropsis tasmanica* Baly, 1866	151	16	8	5	21.9
*Paropsisterna agricola* (Chapuis, 1877)	1279	23*	11	4	27.0
*Paropsisterna bimaculata* (Olivier, 1807)	281	10*	5	3	0.7
*Paropsisterna decolorata*(Chapuis, 1877)	83	12	8	1	0
*Paropsisterna morio* (Fabricius, 1787)	6	1	1	1	0
*Paropsisterna nobilitata* (Erichson, 1842)	98	11	7	3	2.0
Paropsisterna variicollis* (Chapuis 1877)	780	26	12	4	30.1

Since paropsine beetles lay eggs in batches, number of independent collections refers to the number of differing larval groupings collected to maximize the chance of finding *Eadya* parasitism.

* Indicates a minimum as some independent collections were pooled.

**Table 4:** Data from sentinel trials, including parasitism rates of recovered sentinels by *Eadya* and Tachinidae following 72 hours in the field.

Location, Year	Sentinel Beetle Host	% Parasitized by *Eadya*	% Parasitized by Tachinidae	Total larvae recovered
**Moina, 2011**	*P*. *charybdis*	3.3	0	30
	*Pst*. *agricola*	0	2.8	72
	*Pst*. *selmani*	11.8	7.9	76
**Ellendale, 2012**	*P*. *charybdis*	5.0	4.1	121
	*Pst*. *agricola*	6.0	9.6	83
**Moina, 2012**	*P*. *charybdis*	3.1	29.0	174
	*Pst*. *agricola*	12.9	20.9	158
**Runnymede, 2012**	*P*. *charybdis*	6.0	0	50
**Mount Nelson, 2012**	*P*. *charybdis*	6.3	0	94
**The Lea, 2012**	*P*. *charybdis*	0	0	66

### Species delimitation in *Eadya*

**Fig 3:**
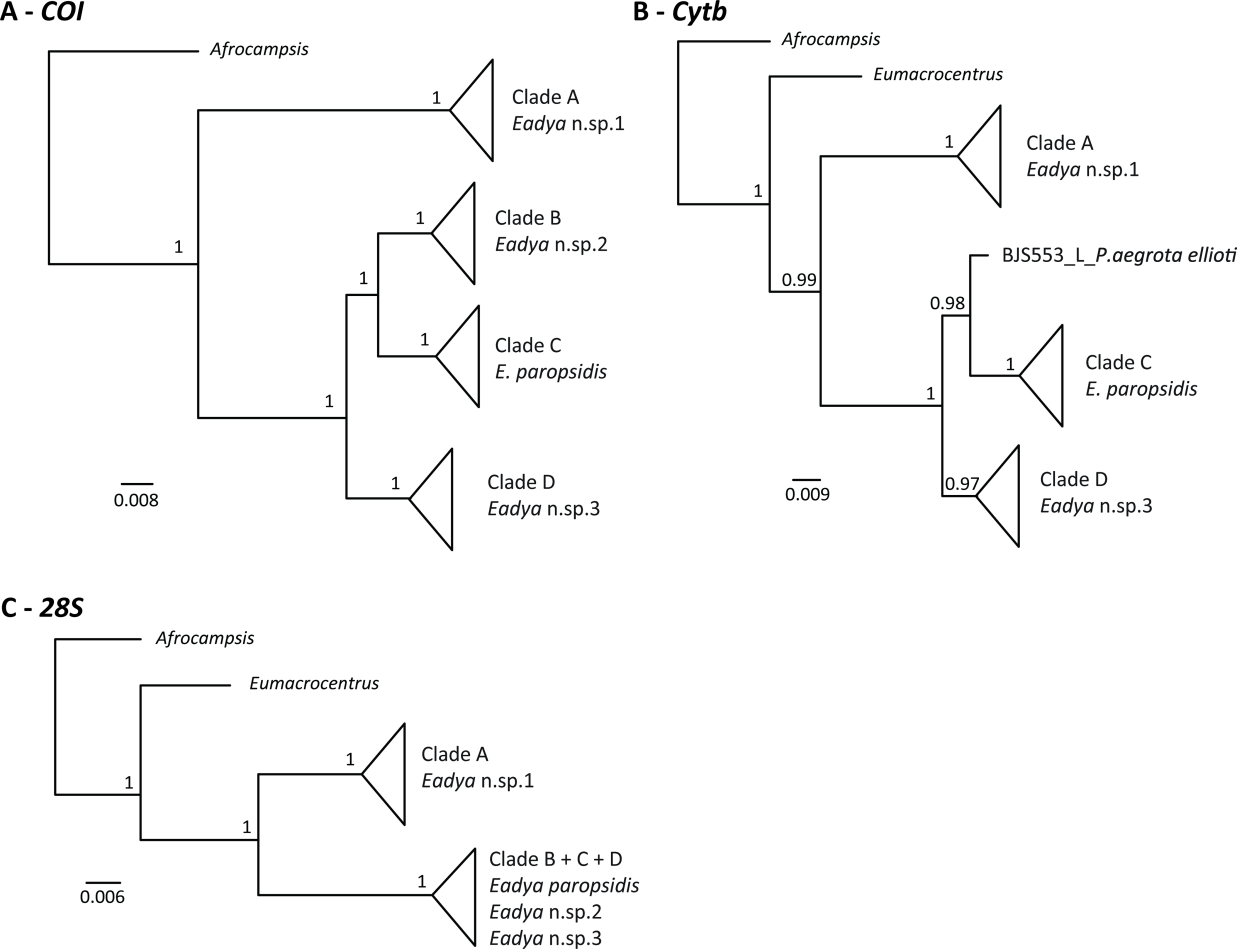
Bayesian analysis of individual gene trees with major clades collapsed based on a phylogenetic species concept. Posterior probabilities are listed near the relevant nodes. Clades and corresponding putative species are labeled. Scale bars refer to number of substitutions for tree branches. **A**. *Cytochrome oxidase I* (*COI*) mtDNA. **B**. *Cytochrome B* (*Cytb*) mtDNA. **C**. *28S* rRNA D1-D3 region.

For *COI*, a total of 672 characters and 177 taxa were included in the analysis, including one outgroup. Four distinct clades were recovered and well supported (pp = 1.0) (Figs 3A and S2) and assigned as putative species based on a phylogenetic species concept (monophyly with high support) [59, 60] and a distinct barcoding gap (greater interclade distance than intraclade distance) [61]. Of the delineated morphospecies, *Eadya* sp. 1 corresponded to Clade A, *Eadya* sp. 2 to Clade B, *E*. *paropsidis* to Clade C and *Eadya* sp. 3 to Clade D (Fig 3A). Clade D had some interclade structure, labeled Clade D1 and D2 (S2 Fig). We consider both of these clades to correspond to *Eadya* sp. 3 because: (1) there are no amino acid differences between sequences of the members of these two clades; (2) the genetic distance between the two clades was only 1.1%; and (3) the two clades were not well supported. Thus, our four delineated morphospecies correspond perfectly to the four phylogenetic species delineated with *COI*. The average interclade genetic distances between all putative phylospecies of *Eadya* (Clades A-D) ranged from 8.7% to 31.2% (Table 5A), well above typical DNA barcoding thresholds (~2–3%) for species delimitation [62, 14], including in Braconidae [49, 19]. There was also very low average intraclade variation, with most clades exhibiting less than 1% genetic distance across all taxa, even though specimens within clades were sampled from different hosts, localities, and across different years (Table 1 and Table 5A).

**Table 5:** Average interspecific and intraspecific genetic distances for *COI* for: A, *Eadya* species; B, beetle species.

**A**	*E*. *paropsidis*	*E*. sp. 1	*E*. sp. 2	*E*. sp. 3						
*E*. *paropsidis*	0.0%									
*E*. sp. 1	31.2%	0.5%								
*E*. sp. 2	8.7%	30.5%	1.1%							
*E*. sp. 3	10.0%	31.1%	10.3%	0.2%						
**B**	*P*. *charybdis*	*P*. *tasmanica*	*Pst*. *agricola*	*Pst*. *beata*	*Pst*. *bimaculata*	*Pst*. *decolorata*	*Pst*. variicollis* (TAS)	*Pst*. variicollis* (NZL)	*Pst*. variicollis* complex
*P*. *aegrota elliotti*									
*P*. *charybdis*	0.3%								
*P*. *tasmanica*	18.8%	0.0%							
*Pst*. *agricola*	19.7%	19.0%	0.6%						
*Pst*. *beata*	18.7%	17.2%	16.8%	n/a					
*Pst*. *bimaculata*	18.3%	20.1%	11.1%	17.8%	0.2%				
*Pst*. *decolorata*	18.0%	20.4%	10.9%	18.6%	10.2%	0.5%			
*Pst*. variicollis* (TAS)	20.2%	20.1%	10.1%	19.0%	9.2%	11.7%	0.4%		
*Pst*. variicollis* (NZL)	19.2%	17.2%	9.0%	15.9%	9.5%	10.9%	1.6%	0.0%	
*Pst*. variicollis* complex	19.7%	18.7%	9.5%	17.5%	9.3%	11.3%	n/a	n/a	0.9%

Intraspecific distances are highlighted in grey. For beetles, *Pst*. *obovata* and *Pst*. *variicollis* are listed separately and together, as the validity of these taxa as separate species was not confirmed.

**Fig 4:**
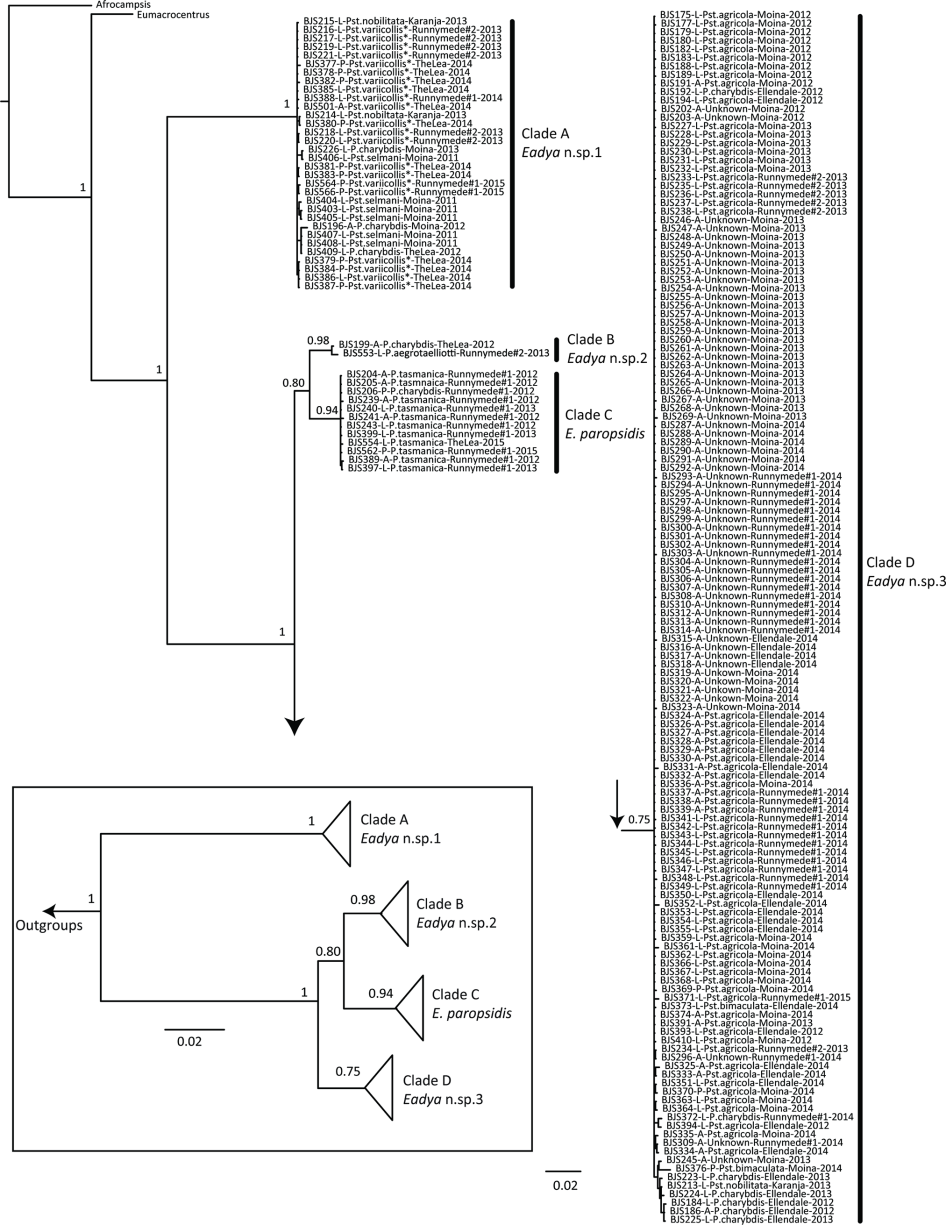
Bayesian analysis of the concatenated dataset with *COI*, *Cytb*, and *28S* combined. Posterior probabilities for major clades are listed near the relevant nodes. Trees connect at the arrows. Clades and corresponding putative species are labeled. Taxon names include voucher numbers, stage of wasp, beetle host name from which the wasps were reared, locality collected, and year of collection, as listed in Table 1. Hosts listed as *Pst*. variicollis* indicate this host is part of a complex of unresolved taxonomic status across southern Australia. The boxed inset has major clades collapsed based on a phylogenetic species concept for ease of viewing relevant clades. Scale bars refer to number of substitutions for tree branches.

For *Cytb*, a total of 429 characters and 173 taxa were included in the analysis, including outgroups. The same four major clades corresponding to the four hypothesized morphospecies were recovered (Fig 3B and S3 Fig) and were well supported (pp ≥ 0.97). Unfortunately, only one taxon from Clade B was amplified for this gene (BJS553 –dissected larval specimen), but this taxon (*Eadya* sp. 2) was still recovered as sister to *E*. *paropsidis*. For *28S*, a total of 893 characters and 162 taxa were included in the analysis and only two well supported clades (pp = 1) were recovered (Fig 3C and S4 Fig). Clade A (*Eadya* sp. 1) was congruent across all genes but all other taxa from *COI* and *Cytb* were recovered in a single clade. As this large Clade contains *Eadya* sp. 2, *E*. *paropsidis*, and *Eadya* sp. 3 (Clades B, C, and D, respectively), *28S* appears to be too conserved for species delimitation in this group. There were limited substitutions across identified morphospecies within the large clade, even within hypervariable regions (RECs, RAAs, and RSCs) that may vary across closely related species [52]. A concatenated dataset was also analyzed with all three genes. The same major clades recovered across *COI* and *Cytb* were also recovered here albeit some with less support, but again supporting four distinct species of *Eadya*, including *E*. *paropsidis* (Fig 4). All taxa are clearly identifiable by morphology, although *E*. *paropsidis* and *Eadya* sp. 3 are very similar morphologically, with *E*. *paropsidis* having a more concave occiput, an emarginate occipital carina, and being larger in size.

### Beetle species identification

**Fig 5:**
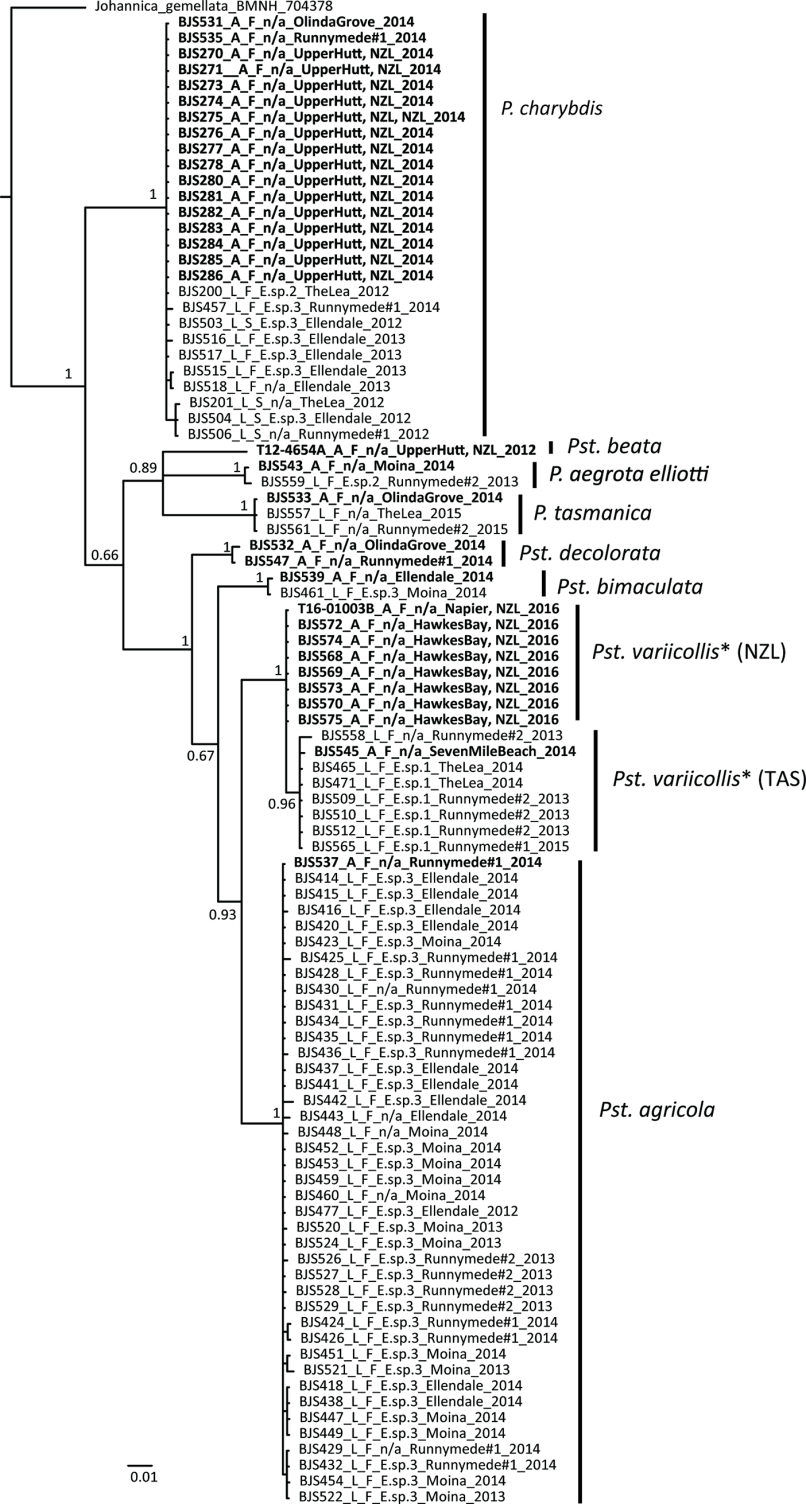
Bayesian analysis of the *COI* dataset for beetle specimens. Posterior probabilities for major clades are listed near the relevant nodes. Adult specimens are in bold. Taxon names include voucher numbers, stage of beetle, method of collection, wasp species name, locality collected in Tasmania (NZL added if collected in New Zealand), and year of collection, as listed in Table 2. Clades are labeled with the identified beetle species based on the placement of the adult reference voucher specimens. Scale bar refers to number of substitutions for tree branches.

*COI* was amplified from beetle remains regardless if the wasp was reared or dissected from the host. Due to degradation of host material, DNA extraction was successful for only 48 parasitized beetles: 35 *Pst*. *agricola*, one *Pst*. *bimaculata*, one *P*. *aegrota elliotti*, seven *P*. *charybdis*, and six specimens identified as *Pst*. variicollis* (Table 2). All putatively identified larval material was recovered in well supported monophyletic clades with the correct adult reference voucher (Fig 5), indicating that larval identifications were accurate. All *Pst*. variicollis* samples were recovered in a strongly supported monophyletic clade. However, there was clade structure with respect to location, such that all *Pst*. variicollis* (from Tasmania) were recovered in a well-supported subclade, indicating distinct differences between samples from New Zealand and Tasmania. There was an average 1.6% genetic distance between the Tasmanian and New Zealand *Pst*. variicollis* (1.6%, Table 5B). Thus, these samples are either from the same species and the different clades are representative of population level differences between New Zealand and Tasmania samples, or they may represent different, but very closely related species. Regardless, the results highlight the need for a revision of *Pst*. variicollis*, which is particularly important as this species was discovered in New Zealand in 2016, representing another potential serious pest to the forest industry [63]. Samples of *P*. *charybdis* from New Zealand were recovered with samples from Tasmania, Australia in a well-supported clade, demonstrating no distinct population level differences between beetles from the two different countries. The average genetic distance among all *P*. *charybdis* was low at 0.3% (Table 5B).

## Discussion

### *Eadya paropsidis* is a complex of species

Based on morphological examination, molecular data from three genes, and host-association data, *Eadya paropsidis* is not a single species, but rather a complex of species. Two of these species are cryptic, with limited morphological characters separating them: *E*. *paropsidis* and *Eadya* sp. 3. Interestingly, these two taxa were suspected to be different species in the original description of *Eadya* by Huddleston and Short (37). They state, “there is a series of eight specimens in ANIC [Australian National Insect Collection] which agree well with *E*. *paropsidis* except that the occiput is less concave, the propodeum is less abruptly divided and the insect smaller. More material is needed to decide if these specimens are succinctly distinct to be described as a new species or merely variants of *E*. *paropsidis* (p. 319).” Our morphological examinations along with molecular analyses confirm that the smaller variant is indeed a new species (*Eady*a sp. 3), and corresponds to Clade D in *COI*, *Cytb*, and the concatenated analysis (Figs 3A, 3B and 5). Although *COI* and *Cytb* were congruent, the D2-D3 region of *28S* was a poor marker for species delimitation, as only two species were delimited from the *28S* phylogeny (Fig 3C). Substitutions in the *28S* regions of ambiguity, where high rates of nucleotide variation are typically found [51] were minimal, ranging from no variation to a few single nucleotide polymorphisms. Genetic distances between species of *Eadya* for *COI* were high, ranging from 8.7 to 31.2%. In particular, *Eadya* sp. 1 had numerous genetic (over 30%) and morphological differences when compared to other species. Pupal cocoon color varied within species with *Eadya* sp. 1 (mostly white), *Eadya* sp. 3 (mostly brown) and *Eadya paropsidis* (mostly white) and was not therefore a reliable aid to species identification. Descriptions of all new species and a key to all species of *Eadya* can be found in Ridenbaugh et al. [43].

### *Eadya* host plasticity

A list of all known host records for all four *Eadya* species is listed in Table 6. All species of *Eadya* can utilize multiple hosts, although some wasps have stronger associations with specific taxa. Two host records were only from sentinels, *E*. *paropsidis* from *P*. *charybdis* and *Eadya* sp. 1 from *Pst*. *selmani* (Table 6). *Eadya* sp. 2 was rarely found and not reared successfully to adulthood in the laboratory. All species of *Eadya* were reared from the target pest, *P*. *charybdis*. *Eadya paropsidis* was largely specific to *P*. *tasmanica* in Tasmania. However, from the original description [37], *E*. *paropsidis* was reared from *P*. *atomaria* in mainland Australia, and several subsequent studies list additional hosts for this species [38, 39]. Considering our findings and by examining morphology of specimens, all *Pst*. *agricola* host records are actually *Eadya* sp. 3 and not *E*. *paropsidis*. This may also be the case for those records for *Pst*. *bimaculata*, though there are no specimens from these earlier records to confirm this.

**Table 6:** Known host relationships for the four species of *Eadya* based on reared beetle records.

*Eadya paropsidis*	*Eadya* sp. 1	*Eadya* sp. 2	*Eadya* sp. 3
-	-	*P*. *aegrota elliotti*	-
*P*. *atomaria*^1^	-	-	-
*P*. *charybdis*^2^	*P*. *charybdis*	*P*. *charybdis*	*P*. *charybdis*
*P*. *tasmanica*	-	-	-
-	-	-	*Pst*. *agricola*
-	-	-	*Pst*. *bimaculata*
-	*Pst*. *nobilitata*	-	*Pst*. *nobilitata*
-	*Pst*. *selmani*^2^	-	-
-	*Pst*. variicollis*	-	-

^1^Australian mainland only from Huddleston $ Short, 1978.

^2^ From sentinel trial only.

*Eadya* sp. 1 and 3 were recovered from multiple hosts. However, *Eadya* sp. 1 was most commonly associated with *Pst*. variicollis* or *Pst*. *selmani* and never from *Pst*. *agricola* or *Pst*. *bimaculata*, while *Eadya* sp. 3 was never reared from *Pst*. variicollis* or *Pst*. *selmani*, demonstrating strong species level differences in host usage despite some cross over in host taxa. *Eadya* sp. 3 almost exclusively used either *Pst*. *agricola* or *P*. *charybdis*, although *Pst*. *bimaculata* and *Pst*. *nobilitata* were rare hosts (Fig 5). Although the majority of *Eadya* sp. 3 collected were from *Pst*. *agricola* hosts, this reflects the relative abundance or availability of this host in our chosen field sites. *Pst*. *agricola* is far more abundant in the plantation locations sampled, whereas *P*. *charybdis* is rare and hard to collect at any location. Reasons for the relative rarity of *P*. *charybdis* in Tasmania are unknown, but we cannot rule out that this species suffers under high natural enemy loadings in Tasmania; *P*. *charybdis* is known to be a host to three species of phoretic mite in Tasmania [64] in addition to egg and larval parasitoids and ladybird predators. Practical difficulties in sampling *P*. *charybdis* also arise due to both adult and larval feeding preferences for flush adult foliage high in the crown (rather than waxy juvenile leaves) of Eucalypts in the subgenus *Symphyomyrtus*. First instar larvae of *P*. *charybdis* tend to scatter and feed singly on outermost branches often high in the crown; whereas, *Pst*. *agricola* feed gregariously on the waxy juvenile foliage within easier reach for sampling. Thus, our sampling may have been influenced by the biology of the beetles.

Host-plasticity is likely beneficial for reproductive success of the wasp. The ability to utilize multiple hosts increases the likelihood of successful parasitism due to the greater availability of resources across habitats [65]. This in turn decreases energy expenditure associated with host seeking. Although beneficial to the wasp, host-plasticity does have some implications for the suitability of these species as classical biological control agents.

### Implications for biological control of *P*. *charybdis*, *Pst*. variicollis*, and other invasive paropsines

Although species of *Eadya* display host plasticity, they appear to be restricted to Paropsine beetles (Chrysomelinae) in two closely related and recently revised [66] genera. These beetles are similar across several biological features, including an overlap of spatial range, similar larval phenology (temporal overlap), and related host plants (externally feeding on foliage of *Eucalyptus* species [67–69, 44]. Additionally, relationships within paropsine beetles are closely linked to host plant usage on eucalypts [70]. Thus, species of *Eadya* are restricted to parasitizing a set of very closely related beetles, both phylogenetically and biologically, despite the ability to successfully parasitize multiple species.

There are no native *Eucalyptus* in New Zealand and all paropsine beetles are invasive pests in that country [34]. Another new paropsine incursion was discovered in 2016, The eucalyptus variegated beetle (*Pst*. variicollis*), which has further increased interest in species of *Eadya* as potential biological control agents [63]. Although there are no records of *Eadya* on beetles in any other genera, host specificity testing has not yet been completed. However, if *Eadya* is found to be host specific to beetles within these two genera, as expected from our results, then *P*. *charybdis* makes an excellent candidate for classical biological control. Withers, Allen [34] already selected a list of candidate species to test *Eadya* for potential non-target effects based on rigorous biological and phylogenetic criteria of native beetles and beneficial weed biological control beetles present in New Zealand.

Based on our data, all recent research [34–36, 71] within this system has been conducted on *Eadya* sp. 3, as opposed to *E*. *paropsidis*. This is a promising as *Eadya* sp. 3 had the most records of parasitism from *P*. *charybdis*, relative to other species. Although there were more records from *Pst*. *agricola*, our sampling biases may have influenced part of this result. As *Pst*. *agricola* is not in New Zealand, there would be no additional resources for *Eadya* sp. 3 to utilize if released in that country, which should promote a successful biological control program. Thus, *Eadya* sp. 3 is the best candidate for importation for control of *P*. *charybdis*. This species was commonly collected across most localities, particularly on the wing, demonstrating a wide geographic range for this species.

*Eadya* sp. 1 could be a suitable candidate for classical biological control of the newly invaded *Pst*. variicollis* in New Zealand. As *Eadya* sp. 1 can attack both *P*. *charybdis* and *Pst*. variicollis*, which could provide an added benefit in the control of both pest species. However, negative impacts due to host competition on *P*. *charybdis* would need to be investigated if both *Eadya* sp. 1 and 3 were to be released. Results from this study indicate a careful population/species level study of the *Pst*. *variicollis* complex is necessary to determine the limits of this species. *Eadya* sp. 1 was also recorded from *Pst*. *selmani*, a Tasmanian paropsine that invaded Ireland in 2007 [72] and is a significant pest of *Eucalyptus* (plantations and cut foliage trade). Now that the *Eadya* species complex has been delimited, the next stage for any of these biological control programs will need to be thorough host specificity testing of the most appropriate *Eadya* species. In the case of *P*. *charybdis* biological control, as was the focus of this study, research will investigate *Eadya* sp. 3 against less closely related non-target beetles present in New Zealand [34].

## Conclusions

For a successful biological control program the biological agent must be correctly identified, particularly in the context of potentially cryptic species complexes. This is essential to ensure an adequate assessment of the biological agent of choice as the host range, biological features, behavior, and potential for control may vary between species within these complexes [e.g. 7, 34]. Prior to this study, it was assumed *E*. *paropsidis* was a single species due to limited taxonomic study on *Eadya*. However, based on our molecular and morphologic data, we now know *E*. *paropsidis* is not just one species, but a complex of species attacking *Eucalyptus*-feeding paropsine beetles in Tasmania. This research has important implications for the forest industry as species of *Eucalyptus* have been imported to numerous countries around the world for their pulp and fiber, and ornamental and oil producing properties.

*Eadya* sp. 3 (formally called *Eadya daenerys* Ridenbaugh 2018) [43] is the most suitable candidate for release in New Zealand to control the eucalyptus tortoise beetle, *P*. *charybdis*. *Eadya* sp. 1 (formally called *Eadya annleckiae* Ridenbaugh 2018) [43] should be examined in future research for potential to control *Pst*. variicollis* in New Zealand and *Pst*. *selmani* in Ireland. However, a comprehensive molecular and morphological review of the taxonomic status of the *Pst*. variicollis* complex is needed. This study represents one of the most comprehensive biological control studies to delimit cryptic species and resolve host relationships through mass rearing, and analysis of morphological and molecular data in relation to hosts of a potential parasitoid biological control agent. It also represents a very successful case of biological control researchers collaborating with taxonomists early in the research pipeline, which is the best way to prevent unintended effects of natural enemy introductions to control pests. Finally, this study also provides the necessary data to create a model system to test theories on biological control and multi-trophic community dynamics in invasion biology with respect to paropsine pests, their host *Eucalyptus* plants, and the suite of primary parasitoids that may regulate their populations.

## Supporting information

S1 Fig Map of collection locations in Tasmania, Australia for the recorded *Eadya* host species of paropsine beetles. Maps were constructed from the authors’ own records as well as those of de Little [22], the Atlas of Living Australia (http://www.ala.org.au) and from the Sustainable Timber Tasmania (Forestry Tasmania) insect collection. For a map of *Pst*. *selmani* distribution see Figure 15 in Reid and de Little [40].
(PDF)

S2 Fig Bayesian analysis of *COI* without clades collapsed. Posterior probabilities are listed near the relevant nodes for major clades. Clades and corresponding putative species are labeled. Taxon names include voucher numbers, stage of wasp, beetle host name from which the wasps were reared, locality collected, and year of collection, as listed in Table 1. Scale bar refers to number of substitutions for tree branches.
(PDF)

S3 Fig Bayesian analysis of *Cytb* without clades collapsed. Posterior probabilities are listed near the relevant nodes for major clades. Clades and corresponding putative species are labeled. Taxon names include voucher numbers, stage of wasp, beetle host name from which the wasps were reared, locality collected, and year of collection, as listed in Table 1. Scale bar refers to number of substitutions for tree branches.
(PDF)

S4 Fig Bayesian analysis of *28S* without clades collapsed. Posterior probabilities are listed near the relevant nodes for major clades. Clades and corresponding putative species are labeled. Taxon names include voucher numbers, stage of wasp, beetle host name from which the wasps were reared, locality collected, and year of collection, as listed in Table 1. Scale bar refers to number of substitutions for tree branches.
(PDF)

S1 Table All material examined for this study, including specimens for morphology and type material. (PDF)

S2 Table Primer sequences used in this study and references for sequences and cycling conditions. (PDF)
