# Supplementary material for: When taxonomy and biological control researchers unite: Species delimitation of Eadya parasitoids (Braconidae) and consequences for classical biological control of invasive paropsine pests of Eucalyptus
Source: PLoS One. 2018 Aug 16;13(8):e0201276. doi: 10.1371/journal.pone.0201276 (PMC6095507; doi:10.1371/journal.pone.0201276)
Supplement: S1 Fig — Maps were constructed from the authors’ own records as well as those of de Little [22], the Atlas of Living Australia (http://www.ala.org.au) and from the Sustainable Timber Tasmania (Forestry Tasmania) insect collection. For a map of Pst. selmani distribution see Figure 15 in Reid and de Little [40]. (PDF) [file pone.0201276.s001.pdf]

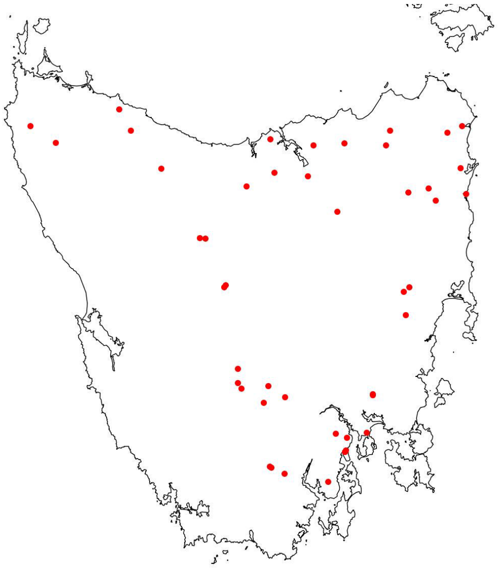

Paropsis aegrota elliotti distribution

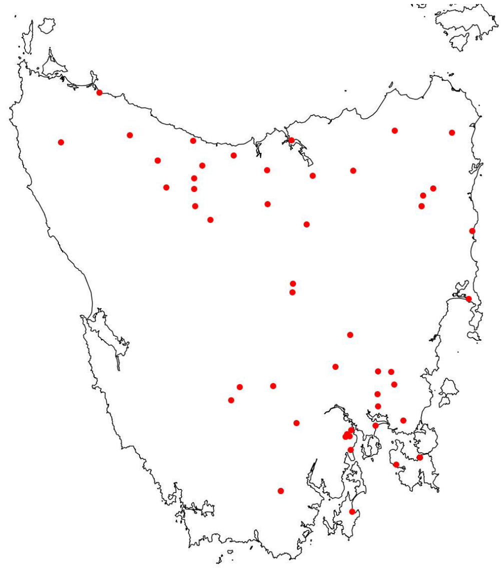

Paropsis charybdis distribution

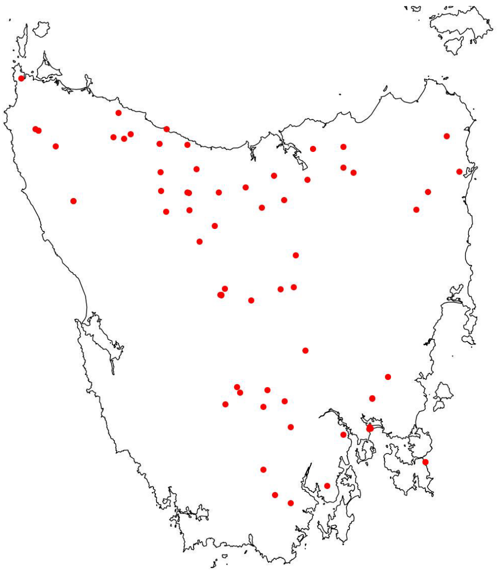

Paropsisterna agricola distribution

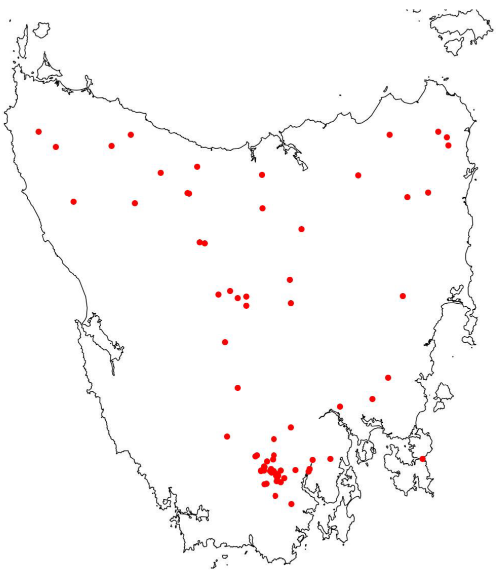

Paropsisterna bimaculata distribution

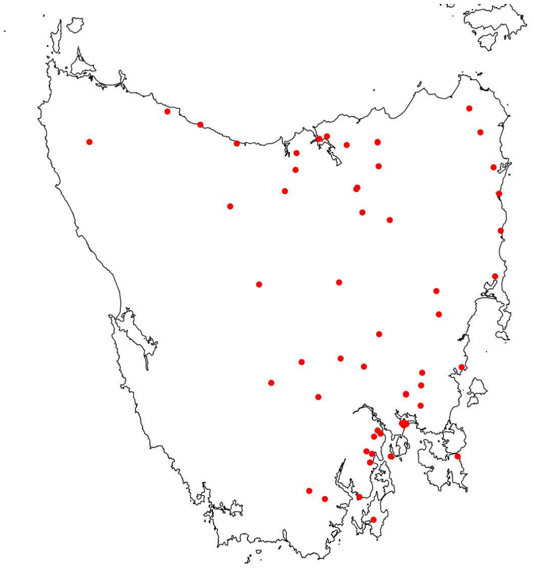

Paropsisterna variicollis distribution

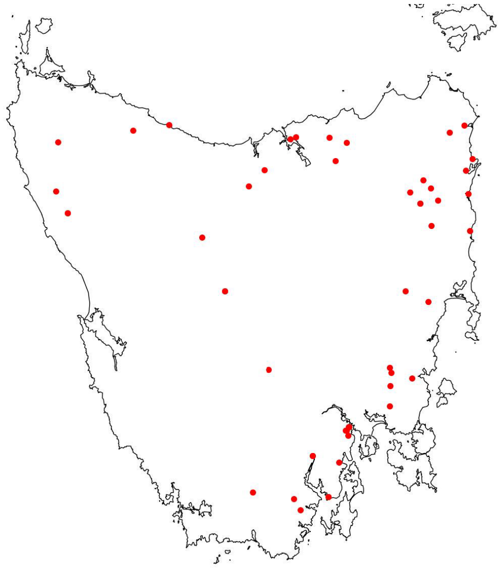

Paropsisterna nobilitata distribution

S1 Fig. Map of collection locations in Tasmania, Australia for the recorded Eadya host species of Paropsine beetles. Maps were constructed from the authors' own records as well as those of de Little [22], the Atlas of Living Australia (http://www.ala.org.au) and from the Sustainable Timber Tasmania (Forestry Tasmania) insect collection. For a map of Pst. selmani distribution see Figure 15 in Reid and de Little [40].
