# Supplementary material for: When taxonomy and biological control researchers unite: Species delimitation of Eadya parasitoids (Braconidae) and consequences for classical biological control of invasive paropsine pests of Eucalyptus
Source: PLoS One. 2018 Aug 16;13(8):e0201276. doi: 10.1371/journal.pone.0201276 (PMC6095507; doi:10.1371/journal.pone.0201276)
Supplement: S4 Fig — Posterior probabilities are listed near the relevant nodes for major clades. Clades and corresponding putative species are labeled. Taxon names include voucher numbers, stage of wasp, beetle host name from which the wasps were reared, locality collected, and year of collection, as listed in Table 1. Scale bar refers to number of substitutions for tree branches. (PDF) [file pone.0201276.s004.pdf]

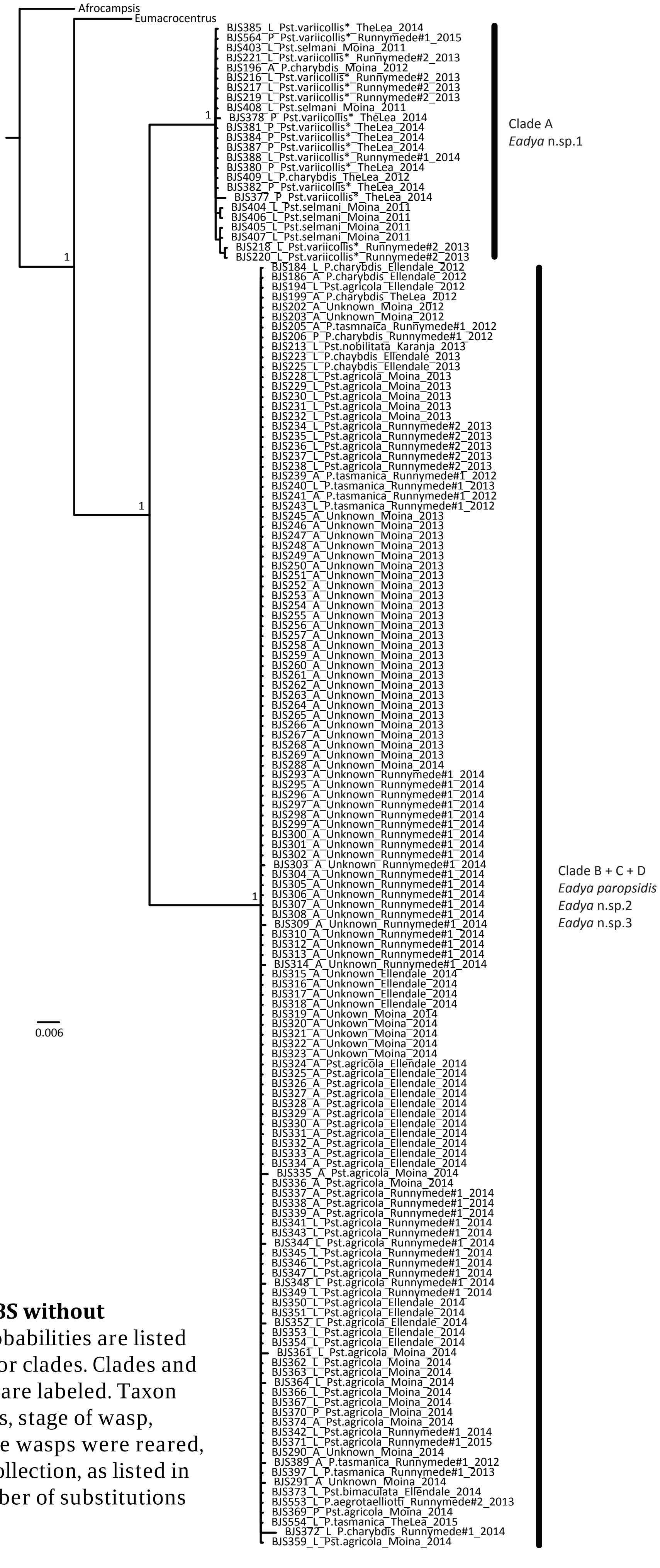

S4 Fig. Bayesian analysis of 28S without clades collapsed. Posterior probabilities are listed near the relevant nodes for major clades. Clades and corresponding putative species are labeled. Taxon names include voucher numbers, stage of wasp, beetle host name from which the wasps were reared, locality collected, and year of collection, as listed in Table 1. Scale bar refers to number of substitutions for tree branches.
