# Supplementary material for: When taxonomy and biological control researchers unite: Species delimitation of Eadya parasitoids (Braconidae) and consequences for classical biological control of invasive paropsine pests of Eucalyptus
Source: PLoS One. 2018 Aug 16;13(8):e0201276. doi: 10.1371/journal.pone.0201276 (PMC6095507; doi:10.1371/journal.pone.0201276)
Supplement: S1 Table — (PDF) [file pone.0201276.s005.pdf]

S1 Table . All material examined for this study, including specimens for morphology and type material.

Wasp Voucher #	Collecting locality	Morpho-species	Phylo-species
BJS204	Runnymede Site #1 TAS; Paropsisterna tasmanica 18.Dec.2012; G.R. Allen; Host larvae collected in field after oviposition observed. Larvae on Eucalyptus ovata	*E. paropsidis*	*E. paropsidis*
BJS205	Runnymede Site #1 TAS; Paropsisterna tasmanica 18.Dec.2012; G.R. Allen; Host larvae collected in field after oviposition observed. Larvae on Eucalyptus ovata	*E. paropsidis*	*E. paropsidis*
BJS206	Runnymede Site #1 TAS; Paropsis charybdis sentinel. Emereged 7.Jan.2013; G.R. Allen		*E. paropsidis*
BJS239	Runnymede Site #1 TAS; Paropsisterna tasmanica 18.Dec.2012; G.R. Allen; Host larvae collected in field after oviposition observed. Larvae on Eucalyptus ovata	*E. paropsidis*	*E. paropsidis*
BJS240	Runnymede Site #1, TAS; Eadya paropsidis larva. Emerged from field collected P. tasmanica. 28 Dec 2013. D. Satchell		*E. paropsidis*
BJS241	Runnymede Site #1 TAS; Paropsisterna tasmanica 18.Dec.2012; G.R. Allen; Host larvae collected in field after oviposition observed. Larvae on Eucalyptus ovata	*E. paropsidis*	*E. paropsidis*
BJS243	Runnymede Site #1 TAS; Paropsisterna tasmanica 18.Dec.2012; G.R. Allen; Host larvae collected in field after oviposition observed. Larvae on Eucalyptus ovata		*E. paropsidis*
BJS389	Runnymede Site #1, TAS; Eadya paropsidis adult dissected from P. tasmanica. 18 Dec 2012. GR. Allen	*E. paropsidis*	*E. paropsidis*
BJS397	Runnymede Site #1, TAS; Eadya paropsidis larva. Emerged from field collected P. tasmanica Pt3. 28 Dec 2013		*E. paropsidis*
BJS399	Runnymede Site #1, TAS; Eadya paropsidis larva. Emerged from field collected P. tasmanica Pt5. 28 Dec 2013		*E. paropsidis*
BJS554	The Lea, TAS; Eadya paropsidis larva. Emerged from field collected P. tasmanica. 3 Dec 2015 T3. GR Allen		*E. paropsidis*
BJS562	Runnymede Site #1, TAS; Eadya paropsidis cocoon. Emerged from P. tasmanica. 22 Dec 2015 TG. GR Allen		*E. paropsidis*
ANIC Paratype	Canberra, A.C.T. 10 January 1954. Dissected from cocoon. Parasite of Paropsis reticulata. CIE COLL No 18079. Eadya paropsidis Paratype ♀ det T. Huddleston, 1977. Aust. Nat. Ins. Coll.	*E. paropsidis*	
UCFC 0 567 693	Runnymede Site #1, TAS. 16 Dec 2015. 42° 38' 11.1" S, 147° 33' 54.7" E. Flying Adult. D Satchell. Female FW4. UCFC 0 567 693.	*E. paropsidis*	
FW2	Runnymede Site #1, TAS. 22 Dec 2015. 42° 38' 08.9" S, 147° 33' 57.9" E. Flying Adult. GR Allen. Male FW2.	*E. paropsidis*	
UCFC 0 567 694	Runnymede Site #1, TAS. 18 Dec 2012. In host, P. tasmanica. GR Allen. TAS1. UCFC 0 567 694.	*E. paropsidis*	
UCFC 0 567 695	Runnymede Site #1, TAS. 22 Dec 2015. 42° 38' 08.9" S, 147° 33' 57.9" E. Flying Adult. GR Allen. Male FW8a. UCFC 0 567 695.	*E. paropsidis*	
ANIC Holotype	18 miles W. of Mogumber, WA. 13 April 1968 I.F.B. Common & M.S. Upton. A39. Eadya falcata Holotype ♀ det. T.Huddleston, 1977. Aust. Nat. Ins. Coll.	*E. falcata*	
ANIC Paratype	18 miles W. of Mogumber, WA. 13 April 1968 I.F.B. Common & M.S. Upton. Eadya falcata Paratype ♂ det. T.Huddleston, 1977. Aust. Nat. Ins. Coll.	*E. falcata*	
BJS196	Moina TAS S41 29.5'E 152 04.7' Paropsis charybdis sentinel; Emerged 2.JAN.2013 G.R. Allen E127	*E. sp.1*	*E. sp.1*
BJS214	Karanja, TAS; Eadya paropsidis larva. Emerged from field collected ? Paropsis nobilitata. 27 Nov 2013. V. Patel		*E. sp.1*
BJS215	Karanja, TAS; Eadya paropsidis larva. Emerged from field collected ? Paropsis nobilitata. 27 Nov 2013. V. Patel		*E. sp.1*
BJS216	Runnymede Site #2, TAS; Eadya paropsidis larva. Emerged from field collected Pst. variicollis*. 27 Dec 2013. S42°40' E147°31'. V. Patel		*E. sp.1*
BJS217	Runnymede Site #2, TAS; Eadya paropsidis larva. Emerged from field collected Pst. variicollis*. 27 Dec 2013. S42°40' E147°31'. V. Patel		*E. sp.1*
BJS218	Runnymede Site #2, TAS; Eadya paropsidis larva. Emerged from field collected Pst. variicollis*. V. Patel		*E. sp.1*
BJS219	Runnymede Site #2, TAS; Eadya paropsidis larva. Emerged from field collected Pst. variicollis*. 27 Dec 2013. S42°40' E147°31'. V. Patel		*E. sp.1*
BJS220	Runnymede Site #2, TAS; Eadya paropsidis larva. Emerged from field collected Pst. variicollis*. 27 Dec 2013. S42°40' E147°31'. V. Patel		*E. sp.1*
BJS221	Runnymede Site #2, TAS; Eadya paropsidis larva. Emerged from field collected Pst. variicollis*. 27 Dec 2013. V. Patel		*E. sp.1*
BJS226	Moina, TAS; Eadya paropsidis larva. Emerged from field collected Paropsis charybdis. 17 Dec 2013.		*E. sp.1*
BJS377	The Lea, TAS #12, Eadya paropsidis cocoon (white). Emerged from Pst. variicollis* 4 Dec 2014		*E. sp.1*
BJS378	The Lea, TAS #12, Eadya paropsidis cocoon (white). Emerged from Pst. variicollis* 4 Dec 2014		*E. sp.1*
BJS379	The Lea, TAS #12, Eadya paropsidis cocoon (white). Emerged from Pst. variicollis* 4 Dec 2014		*E. sp.1*
BJS380	The Lea, TAS #12, Eadya paropsidis cocoon (white). Emerged from Pst. variicollis* 4 Dec 2014		*E. sp.1*
BJS381	The Lea, TAS #12, Eadya paropsidis cocoon (white). Emerged from Pst. variicollis* 4 Dec 2014		*E. sp.1*
BJS382	The Lea, TAS #12, Eadya paropsidis cocoon (white). Emerged from Pst. variicollis* 4 Dec 2014		*E. sp.1*
BJS383	The Lea, TAS #12, Eadya paropsidis cocoon (white). Emerged from Pst. variicollis* 4 Dec 2014		*E. sp.1*
BJS384	The Lea, TAS #12b, Eadya paropsidis cocoon (white). Emerged from Pst. variicollis* 4 Dec 2014		*E. sp.1*
BJS385	The Lea, TAS #12b, Eadya paropsidis larva. Emerged from Pst. variicollis* 4 Dec 2014		*E. sp.1*
BJS386	The Lea, TAS #12b, Eadya paropsidis larva. Emerged from Pst. variicollis* 4 Dec 2014		*E. sp.1*
BJS387	The Lea, TAS #12b, Eadya paropsidis cocoon (white). Emerged from Pst. variicollis* 4 Dec 2014		*E. sp.1*
BJS388	Runnymede Site #1, TAS, Eadya paropsidis larva. Emerged from Pst. variicollis* 12 Feb 2015. GR. Allen		*E. sp.1*
BJS403	Moina, TAS RedWhite1B; Eadya paropsidis larva from Pst. selmani sentinel. 6 Dec 2011. GR. Allen		*E. sp.1*
BJS404	Moina, TAS RedWhite1B(2); Eadya paropsidis larva from Pst. selmani sentinel. 6 Dec 2011. GR. Allen		*E. sp.1*
BJS405	Moina, TAS RedWhite3A; Eadya paropsidis larva from Pst. selmani sentinel. 6 Dec 2011. GR. Allen		*E. sp.1*

BJS406	Moina, TAS RedWhite3A(2); Eadya paropsidis larva from Pst. selmani sentinel. 6 Dec 2011. GR. Allen		*E. sp.1*
BJS407	Moina, TAS RedWhite3A(3); Eadya paropsidis larva from Pst. selmani sentinel. 6 Dec 2011. GR. Allen		*E. sp.1*
BJS408	Moina, TAS RedWhite3A(4); Eadya paropsidis larva from Pst. selmani sentinel. 6 Dec 2011. GR. Allen		*E. sp.1*
BJS409	Moina, TAS Blue1B; Eadya paropsidis larva from P. charybdis sentinel. 6 Dec 2011. GR. Allen		*E. sp.1*
BJS501	The Lea, TAS #12, Eadya paropsidis cocoon (brown). Emerged from Pst. variicollis* 4 Dec 2014	*E. sp.1*	*E. sp.1*
BJS564	Runnymede Site #1, TAS; Eadya paropsidis cocoon. Emerged from Pst. variicollis*. 5 Jan 2016. GR Allen		*E. sp.1*
BJS566	Runnymede Site #1, TAS; Eadya paropsidis cocoon. Emerged from Pst. variicollis*. 5 Jan 2016. GR Allen		*E. sp.1*
FW100	Ellendale, TAS. ♀. 21a. 10 Dec 2014. D Satchell.	*E. sp.1*	
BJS199	The Lea TAS. 11.Dec.2012; Em. 26.Dec.2012; G.R. Allen; Field collected in P. charybdis. E135	*E. sp.2*	*E. sp.2*
BJS553	Runnymede Site #2, TAS; Eadya paropsidis larva. Emerged from field collected P. aegrota elliotti. 11 Dec 2013. GR Allen		*E. sp.2*
FW5	Runnymede Site #1, TAS. 13 Dec 2015. 42° 38' 11.1" S, 147° 33' 54.7" E. Flying Adult. D Satchell. Female.	*E. sp.2*	
BJS175	Moina TAS S41 29.5'E 152 04.7' Dec2012; T.M. Withers		*E. sp.3*
BJS177	Moina TAS S41 29.5'E 152 04.7' Dec2012; T.M. Withers		*E. sp.3*
BJS179	Moina TAS S41 29.5'E 152 04.7' Dec2012; T.M. Withers		*E. sp.3*
BJS180	Moina TAS S41 29.5'E 152 04.7' Dec2012; T.M. Withers		*E. sp.3*
BJS182	Moina TAS S41 29.5'E 152 04.7' Dec2012; T.M. Withers		*E. sp.3*
BJS183	Moina TAS S41 29.5'E 152 04.7' Dec2012; T.M. Withers		*E. sp.3*
BJS184	Ellendale TAS. Paropsis charybdis sentinel trial 11. Dec.2012; Em. 28.dec.2012; G.R. Allen; E53		*E. sp.3*
BJS186	Ellendale TAS. Paropsis charybdis sentinel (correction made on original document) Emereged 28.Dec.2012; G.R. Allen; E54; UCFC 0 567 696.	*E. sp.3*	*E. sp.3*
BJS188	Moina TAS S41 29.5'E 152 04.7' Dec2012; T.M. Withers		*E. sp.3*
BJS189	Moina TAS S41 29.5'E 152 04.7' Dec2012; T.M. Withers		*E. sp.3*
BJS191	Moina TAS S41 29.5'E 152 04.7' Paropsisterna agricola sentinel; 28.Dec.2012; T.M. Withers E69	*E. sp.3*	*E. sp.3*
BJS192	Ellendale TAS. Paropsis charybdis (corrected from original document) sentinel trial 11.Dec.2012; Em. 28-30.Dec.2012; G.R. Allen		*E. sp.3*
BJS194	Ellendale TAS. Paropsisterna agricola sentinel trial 11.Dec.2012; Em. 28-30.Dec.2012; G.R. Allen		*E. sp.3*
BJS202	Moina TAS S41 29.5'E 152 04.7' Dec 2012; T. Withers; Netted flying in the field; UCFC 0 567 697.	*E. sp.3*	*E. sp.3*
BJS203	Moina TAS S41 29.5'E 152 04.7' Dec 2012; T. Withers; Netted flying in the field	*E. sp.3*	*E. sp.3*
BJS213	Karanja, TAS; Eadya paropsidis larva. Emerged from field collected ? Paropsis nobilitata. 27 Nov 2013. V. Patel		*E. sp.3*
BJS223	Ellendale, TAS; Eadya paropsidis larva. Emerged from field collected Paropsis charybdis. 23 Dec 2013. D. Satchell		*E. sp.3*
BJS224	Ellendale, TAS; Eadya paropsidis larva. Emerged from field collected Paropsis charybdis. 23 Dec 2013. D. Satchell		*E. sp.3*
BJS225	Ellendale, TAS; Eadya paropsidis larva. Emerged from field collected Paropsis charybdis. 23 Dec 2013. D. Satchell		*E. sp.3*
BJS227	Moina, TAS; Eadya paropsidis larva. Emerged from field collected Paropsis agricola. 17 Dec 2013.		*E. sp.3*
BJS228	Moina, TAS; Eadya paropsidis larva. Emerged from field collected Paropsis agricola. 17 Dec 2013.		*E. sp.3*
BJS229	Moina, TAS; Eadya paropsidis larva. Emerged from field collected Paropsis agricola. 17 Dec 2013.		*E. sp.3*
BJS230	Moina, TAS; Eadya paropsidis larva. Emerged from field collected Paropsis agricola. 17 Dec 2013.		*E. sp.3*
BJS231	Moina, TAS; Eadya paropsidis larva. Emerged from field collected Paropsis agricola. 17 Dec 2013.		*E. sp.3*
BJS232	Moina, TAS; Eadya paropsidis larva. Emerged from field collected Paropsis agricola. 17 Dec 2013.		*E. sp.3*
BJS233	Runnymede Site #2, TAS; Eadya Paropsidis larva. Emerged from field collected Paropsis agricola. 11 Dec 2013. S42°40' E147°31'		*E. sp.3*
BJS234	Runnymede Site #2, TAS; Eadya Paropsidis larva. Emerged from field collected Paropsis agricola. 11 Dec 2013. S42°40' E147°31'		*E. sp.3*
BJS235	Runnymede Site #2, TAS; Eadya Paropsidis larva. Emerged from field collected Paropsis agricola. 11 Dec 2013. S42°40' E147°31'		*E. sp.3*
BJS236	Runnymede Site #2, TAS; Eadya Paropsidis larva. Emerged from field collected Paropsis agricola. 11 Dec 2013. S42°40' E147°31'		*E. sp.3*
BJS237	Runnymede Site #2, TAS; Eadya Paropsidis larva. Emerged from field collected Paropsis agricola. 11 Dec 2013. S42°40' E147°31'		*E. sp.3*
BJS238	Runnymede Site #2, TAS; Eadya Paropsidis larva. Emerged from field collected Paropsis agricola. 11 Dec 2013. S42°40' E147°31'		*E. sp.3*
BJS245	Moina, TAS. Eadya paropsidis female. 17 Dec 2013.	*E. sp.3*	*E. sp.3*
BJS246	Moina, TAS. Eadya paropsidis female. 17 Dec 2013.	*E. sp.3*	*E. sp.3*
BJS247	Moina, TAS. Eadya paropsidis female. 17 Dec 2013.	*E. sp.3*	*E. sp.3*
BJS248	Moina, TAS. Eadya paropsidis female. 17 Dec 2013.	*E. sp.3*	*E. sp.3*
BJS249	Moina, TAS. Eadya paropsidis female. 17 Dec 2013.	*E. sp.3*	*E. sp.3*
BJS251	Moina, TAS. Eadya paropsidis female. 17 Dec 2013.	*E. sp.3*	*E. sp.3*
BJS252	Moina, TAS. Eadya paropsidis female. 17 Dec 2013.	*E. sp.3*	*E. sp.3*
BJS250	Moina, TAS. Eadya paropsidis female. 17 Dec 2013.	*E. sp.3*	*E. sp.3*
BJS253	Moina, TAS. Eadya paropsidis female. 2 Dec 2013.	*E. sp.3*	*E. sp.3*
BJS254	Moina, TAS. Eadya paropsidis female. 2 Dec 2013.	*E. sp.3*	*E. sp.3*
BJS255	Moina, TAS. Eadya paropsidis female. 2 Dec 2013.	*E. sp.3*	*E. sp.3*
BJS256	Moina, TAS. Eadya paropsidis male. 2 Dec 2013. UCFC 0 567 698.	*E. sp.3*	*E. sp.3*
BJS257	Moina, TAS. Eadya paropsidis male. 2 Dec 2013. UCFC 0 567 699.	*E. sp.3*	*E. sp.3*

BJS258	Moina, TAS. Eadya paropsidis male. 2 Dec 2013. UCFC 0 567 700.	*E. sp.3*	*E. sp.3*
BJS259	Moina, TAS. Eadya paropsidis male. 2 Dec 2013.	*E. sp.3*	*E. sp.3*
BJS260	Moina, TAS. Eadya paropsidis male. 2 Dec 2013.	*E. sp.3*	*E. sp.3*
BJS261	Moina, TAS. Eadya paropsidis male. 2 Dec 2013. UCFC 0 567 701.	*E. sp.3*	*E. sp.3*
BJS262	Moina, TAS. Eadya paropsidis male. 2 Dec 2013. UCFC 0 567 702.	*E. sp.3*	*E. sp.3*
BJS263	Moina, TAS. Eadya paropsidis male. 17 Dec 2013. UCFC 0 567 703.	*E. sp.3*	*E. sp.3*
BJS264	Moina, TAS. Eadya paropsidis male. 17 Dec 2013. UCFC 0 567 704.	*E. sp.3*	*E. sp.3*
BJS265	Moina, TAS. Eadya paropsidis male. 17 Dec 2013. UCFC 0 567 705.	*E. sp.3*	*E. sp.3*
BJS266	Moina, TAS. Eadya paropsidis male. 17 Dec 2013.	*E. sp.3*	*E. sp.3*
BJS267	Moina, TAS. Eadya paropsidis male. 17 Dec 2013.	*E. sp.3*	*E. sp.3*
BJS268	Moina, TAS. Eadya paropsidis male. 17 Dec 2013. UCFC 0 567 706.	*E. sp.3*	*E. sp.3*
BJS269	Moina, TAS. Eadya paropsidis male. 17 Dec 2013. UCFC 0 567 707.	*E. sp.3*	*E. sp.3*
BJS287	Moina, TAS. Eadya paropsidis male. 5 Dec 2014. D. Satchell	*E. sp.3*	*E. sp.3*
BJS288	Moina, TAS. Eadya paropsidis male. 5 Dec 2014. D. Satchell	*E. sp.3*	*E. sp.3*
BJS289	Moina, TAS. Eadya paropsidis male. 5 Dec 2014. D. Satchell	*E. sp.3*	*E. sp.3*
BJS290	Moina, TAS. Eadya paropsidis male. 5 Dec 2014. D. Satchell	*E. sp.3*	*E. sp.3*
BJS291	Moina, TAS. Eadya paropsidis male. 5 Dec 2014. D. Satchell	*E. sp.3*	*E. sp.3*
BJS292	Moina, TAS. Eadya paropsidis female. 5 Dec 2014. D. Satchell. UCFC 0 567 708.	*E. sp.3*	*E. sp.3*
BJS293	Runnymede Site #1, TAS. Eadya paropsidis male. 7 Dec 2014. D. Satchell	*E. sp.3*	*E. sp.3*
BJS294	Runnymede Site #1, TAS. Eadya paropsidis male. 7 Dec 2014. D. Satchell	*E. sp.3*	*E. sp.3*
BJS295	Runnymede Site #1, TAS. Eadya paropsidis male. 7 Dec 2014. D. Satchell. UCFC 0 567 709.	*E. sp.3*	*E. sp.3*
BJS296	Runnymede Site #1, TAS. Eadya paropsidis male. 7 Dec 2014. D. Satchell	*E. sp.3*	*E. sp.3*
BJS297	Runnymede Site #1, TAS. Eadya paropsidis male. 8 Dec 2014. D. Satchell	*E. sp.3*	*E. sp.3*
BJS298	Runnymede Site #1, TAS. Eadya paropsidis male. 8 Dec 2014. D. Satchell	*E. sp.3*	*E. sp.3*
BJS299	Runnymede Site #1, TAS. Eadya paropsidis male. 8 Dec 2014. D. Satchell	*E. sp.3*	*E. sp.3*
BJS300	Runnymede Site #1, TAS. Eadya paropsidis male. 8 Dec 2014. D. Satchell	*E. sp.3*	*E. sp.3*
BJS301	Runnymede Site #1, TAS. Eadya paropsidis male. 8 Dec 2014. D. Satchell. UCFC 0 567 710.	*E. sp.3*	*E. sp.3*
BJS302	Runnymede Site #1, TAS. Eadya paropsidis female. 8 Dec 2014. D. Satchell. UCFC 0 567 711.	*E. sp.3*	*E. sp.3*
BJS303	Runnymede Site #1, TAS. Eadya paropsidis female. 8 Dec 2014. D. Satchell. UCFC 0 567 712.	*E. sp.3*	*E. sp.3*
BJS304	Runnymede Site #1, TAS. Eadya paropsidis female. 8 Dec 2014. D. Satchell. UCFC 0 567 713.	*E. sp.3*	*E. sp.3*
BJS305	Runnymede Site #1, TAS. Eadya paropsidis female. 8 Dec 2014. D. Satchell. UCFC 0 567 714.	*E. sp.3*	*E. sp.3*
BJS306	Runnymede Site #1, TAS. Eadya paropsidis female. 8 Dec 2014. D. Satchell	*E. sp.3*	*E. sp.3*
BJS307	Runnymede Site #1, TAS. Eadya paropsidis male. 9 Dec 2014. D. Satchell	*E. sp.3*	*E. sp.3*
BJS308	Runnymede Site #1, TAS. Eadya paropsidis male. 9 Dec 2014. D. Satchell	*E. sp.3*	*E. sp.3*
BJS309	Runnymede Site #1, TAS. Eadya paropsidis male. 9 Dec 2014. D. Satchell. UCFC 0 567 715.	*E. sp.3*	*E. sp.3*
BJS310	Runnymede Site #1, TAS. Eadya paropsidis male. 9 Dec 2014. D. Satchell	*E. sp.3*	*E. sp.3*
BJS312	Runnymede Site #1, TAS. Eadya paropsidis female. 9 Dec 2014. D. Satchell	*E. sp.3*	*E. sp.3*
BJS313	Runnymede Site #1, TAS. Eadya paropsidis female. 9 Dec 2014. D. Satchell	*E. sp.3*	*E. sp.3*
BJS314	Runnymede Site #1, TAS. Eadya paropsidis female. 9 Dec 2014. D. Satchell	*E. sp.3*	*E. sp.3*
BJS315	Ellendale, TAS. Eadya paropsidis female. 10 Dec 2014. D. Satchell	*E. sp.3*	*E. sp.3*
BJS316	Ellendale, TAS. Eadya paropsidis female. 10 Dec 2014. D. Satchell	*E. sp.3*	*E. sp.3*
BJS317	Ellendale, TAS. Eadya paropsidis female. 10 Dec 2014. D. Satchell	*E. sp.3*	*E. sp.3*
BJS318	Ellendale, TAS. Eadya paropsidis female. 10 Dec 2014. D. Satchell. UCFC 0 567 716.	*E. sp.3*	*E. sp.3*
BJS319	Moina, TAS. Eadya paropsidis male. 11 Dec 2014. D. Satchell	*E. sp.3*	*E. sp.3*
BJS320	Moina, TAS. Eadya paropsidis male. 11 Dec 2014. D. Satchell	*E. sp.3*	*E. sp.3*
BJS321	Moina, TAS. Eadya paropsidis male. 11 Dec 2014. D. Satchell	*E. sp.3*	*E. sp.3*
BJS322	Moina, TAS. Eadya paropsidis male. 11 Dec 2014. D. Satchell	*E. sp.3*	*E. sp.3*
BJS323	Moina, TAS. Eadya paropsidis male. 11 Dec 2014. D. Satchell	*E. sp.3*	*E. sp.3*
BJS324	Ellendale, TAS. Eadya paropsidis adult female. Emerged from field collected Pst. agricola 28e. 10 Dec 2014	*E. sp.3*	*E. sp.3*
BJS325	Ellendale, TAS. Eadya paropsidis adult female. Emerged from field collected Pst. agricola 28e. 10 Dec 2014	*E. sp.3*	*E. sp.3*
BJS326	Ellendale, TAS. Eadya paropsidis adult female. Emerged from field collected Pst. agricola 28e. 10 Dec 2014	*E. sp.3*	*E. sp.3*
BJS327	Ellendale, TAS. Eadya paropsidis adult female. Emerged from field collected Pst. agricola 28e. 10 Dec 2014	*E. sp.3*	*E. sp.3*
BJS328	Ellendale, TAS. Eadya paropsidis adult female. Emerged from field collected Pst. agricola 28e. 10 Dec 2014	*E. sp.3*	*E. sp.3*
BJS329	Ellendale, TAS. Eadya paropsidis adult female. Emerged from field collected Pst. agricola 28e. 10 Dec 2014	*E. sp.3*	*E. sp.3*
BJS330	Ellendale, TAS. Eadya paropsidis adult female. Emerged from field collected Pst. agricola 28e. 10 Dec 2014	*E. sp.3*	*E. sp.3*

BJS331	Ellendale, TAS. Eadya paropsidis adult male. Emerged from field collected Pst. agricola 28e. 10 Dec 2014	*E. sp.3*	*E. sp.3*
BJS332	Ellendale, TAS. Eadya paropsidis adult male. Emerged from field collected Pst. agricola 28e. 10 Dec 2014	*E. sp.3*	*E. sp.3*
BJS333	Ellendale, TAS. Eadya paropsidis adult male. Emerged from field collected Pst. agricola 28e. 10 Dec 2014	*E. sp.3*	*E. sp.3*
BJS334	Ellendale, TAS. Eadya paropsidis adult male. Emerged from field collected Pst. agricola 28e. 10 Dec 2014. UCFC 0 567 718.	*E. sp.3*	*E. sp.3*
BJS335	Moina, TAS. Eadya paropsidis adult male. Emerged from field collected Pst. agricola 33d. 11 Dec 2014	*E. sp.3*	*E. sp.3*
BJS336	Moina, TAS. Eadya paropsidis adult female. Emerged from field collected Pst. agricola 33d. 11 Dec 2014. UCFC 0 567 717.	*E. sp.3*	*E. sp.3*
BJS337	Runnymede Site #1, TAS. Eadya paropsidis adult female. Emerged from field collected Pst. agricola 49a. 9 Dec 2014. UCFC 0 567 719.	*E. sp.3*	*E. sp.3*
BJS338	Runnymede Site #1, TAS. Eadya paropsidis adult female. Emerged from field collected Pst. agricola 50b. 9 Dec 2014. UCFC 0 567 720.	*E. sp.3*	*E. sp.3*
BJS339	Runnymede Site #1, TAS. Eadya paropsidis adult female. Emerged from field collected Pst. agricola 50b. 9 Dec 2014. UCFC 0 567 721.	*E. sp.3*	*E. sp.3*
BJS341	Runnymede Site #1, TAS #47b; Eadya paropsidis larva. Emerged from field collected Pst. agricola. 9 Dec 2014. GR. Allen		*E. sp.3*
BJS342	Runnymede Site #1, TAS #48b; Eadya paropsidis larva. Emerged from field collected Pst. agricola. 9 Dec 2014. GR. Allen		*E. sp.3*
BJS343	Runnymede Site #1, TAS #48c; Eadya paropsidis larva. Emerged from field collected Pst. agricola. 9 Dec 2014. GR. Allen		*E. sp.3*
BJS344	Runnymede Site #1, TAS #50a; Eadya paropsidis larva. Emerged from field collected Pst. agricola. 9 Dec 2014. GR. Allen		*E. sp.3*
BJS345	Runnymede Site #1, TAS #31a; Eadya paropsidis larva. Emerged from field collected Pst. agricola. 9 Dec 2014. GR. Allen		*E. sp.3*
BJS346	Runnymede Site #1, TAS #31a; Eadya paropsidis larva. Emerged from field collected Pst. agricola. 9 Dec 2014. GR. Allen		*E. sp.3*
BJS347	Runnymede Site #1, TAS #31a; Eadya paropsidis larva. Emerged from field collected Pst. agricola. 9 Dec 2014. GR. Allen		*E. sp.3*
BJS348	Runnymede Site #1, TAS #31b; Eadya paropsidis larva. Emerged from field collected Pst. agricola. 9 Dec 2014. GR. Allen		*E. sp.3*
BJS349	Runnymede Site #1, TAS #31b; Eadya paropsidis larva. Emerged from field collected Pst. agricola. 9 Dec 2014. GR. Allen		*E. sp.3*
BJS350	Ellendale, TAS #28a; Eadya paropsidis larva. Emerged from field collected Pst. agricola. 10 Dec 2014. GR. Allen		*E. sp.3*
BJS351	Ellendale, TAS #28a; Eadya paropsidis larva. Emerged from field collected Pst. agricola. 10 Dec 2014. GR. Allen		*E. sp.3*
BJS352	Ellendale, TAS #28a; Eadya paropsidis larva. Emerged from field collected Pst. agricola. 10 Dec 2014. GR. Allen		*E. sp.3*
BJS353	Ellendale, TAS #28b; Eadya paropsidis larva. Emerged from field collected Pst. agricola. 10 Dec 2014. GR. Allen		*E. sp.3*
BJS354	Ellendale, TAS #28c; Eadya paropsidis larva. Emerged from field collected Pst. agricola. 10 Dec 2014. GR. Allen		*E. sp.3*
BJS355	Ellendale, TAS #28c; Eadya paropsidis larva. Emerged from field collected Pst. agricola. 10 Dec 2014. GR. Allen		*E. sp.3*
BJS359	Moina, TAS #33b; Eadya paropsidis larva. Emerged from field collected Pst. agricola. 11 Dec 2014. GR. Allen		*E. sp.3*
BJS361	Moina, TAS #33e; Eadya paropsidis larva. Emerged from field collected Pst. agricola. 11 Dec 2014. GR. Allen		*E. sp.3*
BJS362	Moina, TAS #33g; Eadya paropsidis larva. Emerged from field collected Pst. agricola. 11 Dec 2014. GR. Allen		*E. sp.3*
BJS363	Moina, TAS #35c; Eadya paropsidis larva. Emerged from field collected Pst. agricola. 11 Dec 2014. GR. Allen		*E. sp.3*
BJS364	Moina, TAS #35d; Eadya paropsidis larva. Emerged from field collected Pst. agricola. 11 Dec 2014. GR. Allen		*E. sp.3*
BJS366	Moina, TAS #37b; Eadya paropsidis larva. Emerged from field collected Pst. agricola. 11 Dec 2014. GR. Allen		*E. sp.3*
BJS367	Moina, TAS #37c; Eadya paropsidis larva. Emerged from field collected Pst. agricola. 11 Dec 2014. GR. Allen		*E. sp.3*
BJS368	Moina, TAS #37d; Eadya paropsidis larva. Emerged from field collected Pst. agricola. 11 Dec 2014. GR. Allen		*E. sp.3*
BJS369	Moina, TAS #33a; Eadya paropsidis cocoon (white). Emerged from field collected Pst. agricola. 11 Dec 2014. GR. Allen		*E. sp.3*
BJS370	Moina, TAS #33d; Eadya paropsidis cocoon (white). Emerged from field collected Pst. agricola. 11 Dec 2014. GR. Allen		*E. sp.3*
BJS371	Runnymede Site #1, TAS; Eadya paropsidis larva. Emerged from field collected Pst. agricola 12 Feb 2015. GR. Allen		*E. sp.3*
BJS372	Runnymede Site #1, TAS #18; Eadya paropsidis larva. Emerged from field collected P. charybdis. 9 Dec 2014. GR. Allen		*E. sp.3*
BJS373	Ellendale, TAS #27c; Eadya paropsidis larva. Emerged from field collected Pst. bimaculata. 10 Dec 2014. GR. Allen		*E. sp.3*
BJS374	Moina, TAS. Eadya paropsidis adult male. Emerged from field collected Pst. agricola/charybdis? 35a. 11 Dec 2014	*E. sp.3*	*E. sp.3*
BJS376	Moina, TAS #42; Eadya paropsidis cocoon (white). Emerged from field collected Pst. bimaculata. 11 Dec 2014. GR. Allen		*E. sp.3*
BJS391	Moina, TAS; Eadya paropsidis female adult dissected from Pst. agricola. Pa27 17 Dec 2013	*E. sp.3*	*E. sp.3*
BJS393	Ellendale, TAS; Eadya paropsidis larva. Emerged from field collected Pst. agricola E117. 11 Dec 2012		*E. sp.3*
BJS394	Ellendale, TAS; Eadya paropsidis larva. Emerged from field collected Pst. agricola E118. 11 Dec 2012		*E. sp.3*
BJS410	Moina, TAS E71; Eadya paropsidis larva from Pst. agricola sentinel. 6 Dec 2012		*E. sp.3*
Pin #8	Frankford, TAS. ♀. 3 Dec 2001. AD Rice. NT#5. Pin #8.	*E. sp.3*	
UCFC 0 567 722	Moina, TAS; S41 29.5' E152 04.7'; netted flying in field. Dec. 2012; T Withers. UCFC 0 567 722.	*E. sp.3*	
UCFC 0 567 723	Frankford, TAS. 2 Jan 2002. Malaise Trap. AD Rice, MT6. UCFC 0 567 723.	*E. sp.3*	
UCFC 0 567 724	Moina, TAS; S41 29.5' E152 04.7'; netted flying in field. Dec. 2012; T Withers. UCFC 0 567 724.	*E. sp.3*	
UCFC 0 567 725	Frankford, TAS. 2 Jan 2002. Malaise Trap. AD Rice, MT6. UCFC 0 567 725.	*E. sp.3*	
UCFC 0 567 726	Runnymede TAS, 24 Nov 2015. 42 38' 13.3"S 147 33' 53.8"E. malaise trap GR Allen ♂. MTM9. UCFC 0 567 726.	*E. sp.3*	
UCFC 0 567 727	Runnymede TAS, 16 Dec 2015. 42 38' 08.9"S 147 33' 57.9"E. flying adult GR Allen ♂. M87. UCFC 0 567 727.	*E. sp.3*	
UCFC 0 567 728	Runnymede TAS, 24 Nov 2015. 42 38' 13.3"S 147 33' 53.8"E. malaise trap GR Allen ♂. MTM7. UCFC 0 567 728.	*E. sp.3*	
UCFC 0 567 729	Runnymede TAS. E130. pup 7 Jan 2013. P. charybdis sentinal GR Allen. UCFC 0 567 729.	*E. sp.3*	
MTM2	Runnymede TAS, 24 Nov 2015. 42 38' 13.3"S 147 33' 53.8"E. malaise trap GR Allen ♂. MTM2.	*E. sp.3*	
UCFC 0 567 730	Frankford TAS ♂. 19 Nov 2001. AD Rice NT#4. Pin #12. UCFC 0 567 730.	*E. sp.3*	
EM2	Ellendale TAS ♂. 14 Dec 2015. D Satchell. EM2.	*E. sp.3*	

UCFC 0 567 731	Frankford, TAS. 2 Jan 2002. Malaise Trap, AD Rice. MT6.	*E. sp.3*
UCFC 0 567 732	Moina, TAS; S41 29.5' E152 04.7'; netted flying in field. Dec. 2012; T Withers. UCFC 0 567 732.	*E. sp.3*
Pin #11	Frankford TAS ♂. 19 Nov 2001. AD Rice NT#5. Pin #11.	*E. sp.3*
MTM32	Runnymede TAS. 9 Dec 2015. 42 38' 13.3"S, 147 33' 53.8'E. malaise trap. GR Allen ♂. MTM32.	*E. sp.3*
MTM10	Runnymede TAS, 24 Nov 2015. 42 38' 13.3"S 147 33' 53.8"E. malaise trap GR Allen ♂. MTM10.	*E. sp.3*
MTM14	Runnymede TAS, 24 Nov 2015. 42 38' 13.3"S 147 33' 53.8"E. malaise trap GR Allen ♂. MTM14.	*E. sp.3*
EM1	Ellendale TAS ♂. 14 Dec 2015. D Satchell. EM1.	*E. sp.3*
MTM1	Runnymede TAS, 24 Nov 2015. 42 38' 13.3"S 147 33' 53.8"E. malaise trap GR Allen ♂. MTM1	*E. sp.3*
ANIC Variant #1	The Creel, Kosciusko NSW. 8 Nov 1961. EF Riek. A35. Eadya ? sp, near paropsidis. det T. Huddleston, 1977. Aust Nat. Ins. Coll.	*E. sp.3*
ANIC Variant #2	Canberra ACT. 19 Nov 1958. EF Riek. A34. Eadya ? sp, near paropsidis. det T. Huddleston, 1977. Aust. Nat. Ins. Coll.	*E. sp.3*
ANIC Variant #3	Canberra ACT. 26 Nov 1959. EF Riek. Eadya ? sp, near paropsidis. det T. Huddleston, 1977. Aust. Nat. Ins. Coll.	*E. sp.3*
ANIC Variant #4	Canberra ACT. 26 Nov 1959. EF Riek. Eadya ? sp, near paropsidis. det T. Huddleston, 1977. Aust. Nat. Ins. Coll.	*E. sp.3*
ANIC Variant #5	Canberra ACT. 30 Nov 1959. EF Riek. Eadya ? sp, near paropsidis. det. T. Huddleston, 1977. Aust. Nat. Ins. Coll.	*E. sp.3*
ANIC Variant #6	Canberra ACT. 18 Nov 1960. EF Riek. Eadya ? sp, near paropsidis. det. T. Huddleston, 1977. Aust. Nat. Ins. Coll.	*E. sp.3*
ANIC Variant #7	Canberra ACT. 24 Nov 1960. EF Riek. A35. Eadya ? sp, near paropsidis. det. T. Huddleston, 1977. Aust. Nat. Ins. Coll.	*E. sp.3*
ANIC Variant #8	Black Mt. F.C.T. (ACT). 10 XI 30. W. Broce. Eadya ? sp, near paropsidis. det. T. Huddleston, 1977. Aust. Nat. Ins. Coll.	*E. sp.3*
Pin #3	Frankford TAS ♀. 27 Nov 2000. AD Rice. Em Trap #1. Pin #3.	*E. sp.3*
UCFC 0 567 733	Frankford TAS ♀. 7 Nov 2000. AD Rice. Em Trap #1. Pin #1. UCFC 0 567 733.	*E. sp.3*
UCFC 0 567 734	Runnymede TAS ♀ R1. 8 Dec 2014. flying adult D Satchell. UCFC 0 567 734.	*E. sp.3*
UCFC 0 567 735	Frankford. TAS 2 Jan 2002. Malaise Trap, AD Rice. MT6. UCFC 0 567 735.	*E. sp.3*
UCFC 0 567 736	Frankford TAS ♀. 19 Nov 2001. AD Rice. NT#5. Pin #5. UCFC 0 567 736.	*E. sp.3*
UCFC 0 567 737	Frankford TAS ♀. 3 Dec 2001. AD Rice NT#5. Pin #9. UCFC 0567 737.	*E. sp.3*
UCFC 0 567 738	Frankford TAS ♀. 3 Dec 2001. AD Rice NT#5. Pin #10. UCFC 0567 738.	*E. sp.3*
UCFC 0 567 739	Runnymede TAS ♀ R2. 9 Dec 2014. flying adult D Satchell. UCFC 0 567 739.	*E. sp.3*
UCFC 0 567 740	Frankford TAS ♀. 14 Nov 2001. AD Rice. NT#5. Pin #4. UCFC 0 567 740.	*E. sp.3*
UCFC 0 567 741	Frankford TAS ♀. 19 Nov 2001. AD Rice. NT#5. Pin #7. UCFC 0 567 741.	*E. sp.3*
UCFC 0 567 742	Frankford TAS ♀. 19 Nov 2001. AD Rice. NT#5. Pin #6. UCFC 0 567 742.	*E. sp.3*
UCFC 0 567 743	Frankford, TAS 2 Jan 2002. Malaise Trap. AD Rice. MT6. UCFC 0 567 743.	*E. sp.3*
UCFC 0 567 744	Frankford TAS ♀. 27 Nov 2000. AD Rice. Em Trap #1. Pin #2. UCFC 0567 744.	*E. sp.3*
UCFC 0 567 745	Frankford, TAS 2 Jan 2002. Malaise Trap. AD Rice. MT6. UCFC 0 567 745.	*E. sp.3*
A.E.I. Sep/05	King William Range, I. 8-23. Tasmania. Eadya. American Entomological Institute Sep/05.	*E. sp.3*
ANIC E. n.sp.4	Upper Kangaroo Valley. Nov 24 1960. EF Riek NSW. Eadya sp. det T. Huddleston 1977. A44. Aust. Nat. Ins. Coll.	*E. sp.4*
