# Supplementary material for: When taxonomy and biological control researchers unite: Species delimitation of Eadya parasitoids (Braconidae) and consequences for classical biological control of invasive paropsine pests of Eucalyptus
Source: PLoS One. 2018 Aug 16;13(8):e0201276. doi: 10.1371/journal.pone.0201276 (PMC6095507; doi:10.1371/journal.pone.0201276)
Supplement: S2 Table — (PDF) [file pone.0201276.s006.pdf]

S2 Table. Primer sequences used in this study and references for sequences and cycling conditions.

Gene	Primer Name	Primer Sequence (5' to 3')	PCR Cycling Conditions	Source
28S	28SD1shortF	GUG GUA AAC UCC AUC UAA G	See Sharanowski et al. (2011)	Sharanowski et al. (2011)
	28SD2shortR	ACA TGT TAG ACT CCT TGG TC		
COI	COI-LCO1490	GGT CAA CAA ATC ATA AAG ATA TTG G	See. Schulmester et al. (2002)	Folmer et al. (1994)
	CO1-HCO2198	TAA ACT TCA GGG TGA CCA AAA AAT CA		
CytB	CytB-F	TCT TTT TGA GGA GCW ACW GTW ATT AC	See Belshaw and Quicke (1997)	Belshaw and Quicke (1997)
	CytB-R	AAT TGA ACG TAA AAT WGT RTA AGC AA		
